# Optimization, implementation, and performance of TMS coils with maximum focality and various stimulation depths

**DOI:** 10.1088/1741-2552/ae4382

**Published:** 2026-03-02

**Authors:** Luis J Gomez, David L K Murphy, Lari M Koponen, Rena Hamdan, Yiru Li, Eleanor Wood, Jacob Golden, Noreen Bukhari-Parlakturk, Stefan M Goetz, Angel V Peterchev

**Affiliations:** 1 Department of Electrical and Computer Engineering, Purdue University, West Lafayette, IN 47906, Unites States of America; 2 Department of Psychiatry and Behavioral Sciences, Duke University, Durham, NC 27710, Unites States of America; 3 Department of Neurology, Duke University, Durham, NC 27710, Unites States of America; 4 Department of Electrical and Computer Engineering, Duke University, Durham, NC 27708, Unites States of America; 5 Department of Neurosurgery, Duke University, Durham, NC 27710, Unites States of America; 6 Department of Biomedical Engineering, Duke University, Durham, NC 27708, Unites States of America

**Keywords:** transcranial magnetic stimulation, TMS, coil, focal, depth, energy, integer linear programming optimization

## Abstract

*Objective.* Conventional transcranial magnetic stimulation (TMS) coils generate a diffuse and shallow electric field (E-field) in the brain, resulting in limited spatial targeting precision (focality). Previously, we developed a methodology for designing theoretical TMS coils to achieve maximal focality for a given E-field penetration depth and minimize the required energy. This paper presents the practical design, implementation, and characterization of such focal-deep TMS (fdTMS) coils. *Approach.* We considered how the coil’s shape affects energy requirements and designed a curved ‘hat’ former that enables a wide range of coil placements while improving energy efficiency compared to flat formers. To improve energy efficiency, we introduced optimized-coverage partial-multi-layer windings of the coil. Through simulations with a spherical head model, we benchmarked the focality of the fdTMS E-field in the brain and the scalp, as well as the required energy, against conventional TMS coils. We then implemented two fdTMS coil designs with copper wire wound inside a 3d-printed plastic former. *Main results.* The E-field of the prototype fdTMS coils and conventional figure-8 counterparts were simulated in spherical and realistic head models and measured with a robotic probe, confirming a more compact fdTMS E-field. The fdTMS coils were also compared to two commercial coils with motor mapping in nine human subjects, which confirmed improved focality of fdTMS at the cost of greater E-field spread in the scalp, increased energy loss and heating from the smaller wire diameter and additional windings, and positioning constraints of the curved coil surface. *Significance.* The study findings inform TMS coil implementation for precise mapping and targeting applications, and the design framework can be leveraged for future coil optimizations.

## 1. Introduction

Transcranial magnetic stimulation (TMS) is a non-invasive method for brain stimulation widely employed in neuroscience to investigate and probe brain function and connectivity. Furthermore, TMS has received FDA approval for treating depression [1] as well as various other psychiatric and neurological disorders [2, 3]. During a TMS session, a coil placed on the scalp and driven by brief strong current pulses induces an electric field (E-field) in the brain. Enhancing the focality and depth of this induced brain E-field provides greater flexibility and selectivity in targeting deep brain regions. Consequently, prior studies have attempted to design coils with improved focus and penetration depth [4–19]. We previously developed a computational method for designing focal-deep (fdTMS) coils that achieve optimal trade-offs between focality, depth, and energy. Numerical studies demonstrated that coils designed using this framework can outperform the state-of-the-art figure-8 coils [20]. In this paper, we extend the fdTMS framework beyond its original numerical formulations by explicitly addressing practical implementation constraints. We present an optimization pipeline that turns theoretical windings into manufacturable coil geometries with adequate clearance for wire cross-sections and hybrid-layer builds, improving energy efficiency. We then benchmark the resulting designs against commercial figure-8 coils, showing superior depth–focality trade-offs. E-field simulations and bench experiments confirm that the optimized coils deliver significantly greater focality while remaining feasible for high-current TMS operation.

Figure-8 type coils consist of two circular coils placed side-by-side [21]. Until recently, these coils were considered to provide an optimal depth–focality trade-off [21]. Computational coil design methods employing the stream function approach [22] have recently been used to design coils that improve energy efficiency [11, 19, 23–32], reduce sound artifact [33], and produce more focused and deeply penetrating E-fields [11, 20]. These studies have also revealed that to achieve a more focal stimulation, increased coil energy is required [11]. Additionally, coil supports that better conform to the head will better achieve energy trade-offs than non-conformal ones [11, 23]. However, perfectly conformed coils cannot be used across individual head shapes or different cortical targets, leading to ‘hat’ shaped coils [23]. This study describes an optimized hat-shaped coil support that outperforms flat supports in energy efficiency while providing ergonomic adaptability across head sizes, cranial morphologies, and scalp positions.

The half-maximum E-field threshold—defined relative to the peak and therefore scale-independent—is commonly used as a figure of merit for benchmarking coil focality and stimulation depth [21]. We tested the sensitivity of this choice by analyzing alternative thresholds and by designing coils to optimize depth–focality trade-offs under these thresholds. Designs based on the half-maximum criterion were nearly indistinguishable from those based on other thresholds, indicating that the half-maximum region is a robust figure of merit for ranking coils by E-field distribution shape despite reasonable variations in activation threshold.

The optimized fdTMS coils concentrate smaller windings near the center and adopt more intricate patterns than conventional figure-8 designs. A typical fdTMS coil comprises a compact figure-8 core augmented by four ‘cancellation’ loops and two larger lateral ‘biasing’ loops. Because the figure-8 loops are small and the wire cross-section is constrained for energy efficiency, meeting driver-compatible inductance would otherwise require multilayer windings that increase energy demand. We developed a winding-synthesis method that applies multiple layers only in critical regions and single layers elsewhere, limiting the energy penalty while achieving the target inductance. We also created a semi-automatic workflow to generate a 3D coil former with integrated grooves for the windings and features that enhance subject safety and comfort. Performance was quantified and benchmarked against commercial coils using E-field simulations, bench measurements, and human motor mapping.

In summary, this paper contributes (i) a manufacturability-aware pipeline that converts Pareto-optimal surface currents into realizable multilayer windings under explicit constraints on wire cross-section, winding spacing, and inductance; (ii) a hybrid multilayer discretization strategy that preserves the targeted intracranial E-field while satisfying manufacturing constraints; (iii) a ‘hat’ support that improves placement flexibility over practical cortical targets while remaining partially conformal; and (iv) experimental validation including E-field mapping and human motor mapping, which has not been previously demonstrated for focal-deep designs derived from optimal-current theory.

## 2. Methods and materials

In this section, we describe our proposed approach for designing and fabricating fdTMS coils. Firstly, we introduce the coil design parameters and figures of merit used to assess coil performance. Detailed methodologies for optimizing these parameters were previously presented [20]. Secondly, we describe the methods used to modify the optimal design, making it compatible with our coil fabrication procedures. In the third part, we outline the procedures for coil fabrication and provide details about the validation measurements of the coil’s E-field. Lastly, we provide additional details about figures of merit used for computational comparisons with conventional coils.

### 2.1. Coil design parameters

**Figure 1.**
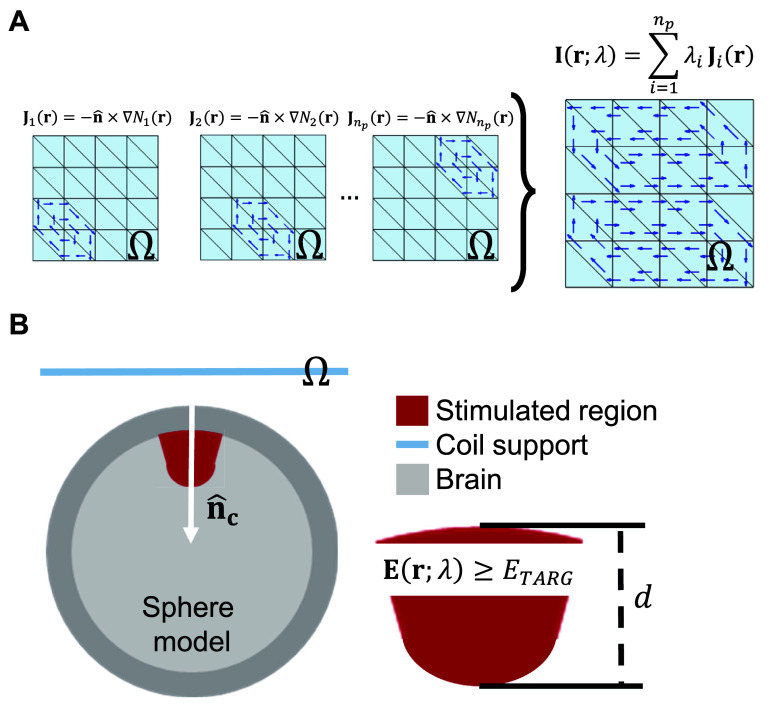
Coil current density parameters and E-field figures of merit definition. (A) Each seed current corresponding to internal nodes of the coil support triangle mesh is linearly combined to generate surface current distributions. (B) The E-field generated in a spherical head model by each coil is computed, and stimulation depth and volume figures of merit are extracted.

The coil windings were assumed to reside on a surface ${{\Omega }}$ that is approximated by a triangle mesh consisting of ${n_p}$ nodes and ${n_t}$ flat triangle cells. The winding paths were chosen such that when driven by a TMS coil driver they will approximate the E-field generated by a surface current on ${{\Omega }}$ (this process is given in the next section). To determine the optimal current, it was assumed that it is in the span of the ${n_p}$ seed currents ${{\mathbf{J}}_i}\left( {\mathbf{r}} \right) = - \widehat {\mathbf{n}} \times \nabla {N_i}\left( {\mathbf{r}} \right)$, where $i {\text{ = 1,2,}} \ldots {\mathrm{,}}{n_p}$, ${\text{ }}{\mathbf{r}} = \left( {x,y,z} \right)$ denotes Cartesian position, and ${N_i}\left( {\mathbf{r}} \right)$ are nodal finite elements on the triangle mesh [22]. In other words, surface current distributions ${\mathbf{I}}\left( {{\mathbf{r}},t} \right)$ are defined on a coil surface ${{\Omega }}$ (figure 1) as

\begin{align*} {\mathbf{I}}\left( {{\mathbf{r}},t;\lambda } \right) = p\left( t \right){\mathbf{I}}\left( {{\mathbf{r}};\lambda } \right) = p\left( t \right)\mathop \sum \limits_{i = 1}^{{n_p}} {\lambda _i}{{\mathbf{J}}_i}\left( {\mathbf{r}} \right) \end{align*}

where $\lambda = {\left( {{\lambda _1},{\lambda _2}, \ldots ,{\lambda _{{n_p}}}} \right)^{\mathrm{T}}}$ is a vector of weights, each ${\lambda _i}$ (where ${i = 1,2,} \ldots {\mathrm{,}}{n_p}$) is a real number, and $p\left( t \right) = {\mathrm{sin}}\left( {\omega t} \right)$ and $\omega = 3000{\text{ }}{{\mathrm{s}}^{ - 1}} \cdot 2\pi $. Note that $p\left( t \right)$ was assumed to be time-harmonic to simplify the exposition. However, because of the relatively low-frequency content of TMS pulses, the results apply to other current waveforms as well.

The above seed currents are known to span all piecewise linear currents with zero divergence on the triangle mesh [34], which thereby forms an adequate basis for approximately including all admissible E-fields generated by non-dissipative surface current distributions on ${{\Omega }}$.

### 2.2. Coil performance figures of merit

The fdTMS coil optimization takes as input a triangle mesh (or parametrization) of the surface ${{\Omega }}$ and a set of seed current distributions ${{\mathbf{J}}_i}\left( {\mathbf{r}} \right)$, where ${{i = 1,2,}} \ldots {\mathrm{,}}{n_p}$. Then, it finds Pareto optimal currents ${\mathbf{I}}\left( {{\mathbf{r}},t;{\lambda _{{\mathrm{opt}}}}} \right)$ that achieve optimal trade-offs with respect to stimulation energy, depth, and volume. The coil figures of merit are defined as follows:

(i) *Minimum stimulation volume*: the stimulated volume $V$ was defined as
  \begin{align*} V \left(\lambda\right)=\int\int\int_{r \epsilon B rain} u\left(\| {{\mathbf{E}}\left( {{\mathbf{r}};{{\lambda }}} \right\|) - {E_{TARG}}} \right) d^3 \mathbf{r}\end{align*}
  where ${\mathbf{E}}\left( {{\mathbf{r}};\lambda } \right)$ denotes peak E-field at location ${\mathbf{r}}$ induced by the surface current ${\mathbf{I}}\left( {{\mathbf{r}},t;\lambda } \right)$, $\left\| \cdot \right\|$ denotes vector magnitude, ${\text{ }}u\left( x \right)$ is a unit step function, the integration is over the brain region (denoted $Brain$), and ${E_{{\mathrm{TARG}}}}$ is the E-field at the targeted depth location. The value of $u\left( {{\mathbf{E}}\left( {{\mathbf{r}};{{\lambda }}} \right) - {E_{TARG}}} \right)$ is one if ${\mathbf{E}}\left( {{\mathbf{r}};{{\lambda }}} \right)$ is above the stimulation threshold and zero otherwise. As a result, equation (2) measures the volume of the region above threshold.
(ii) *Maximum depth of stimulation:* the depth of stimulation $d$ was defined along a line ${\mathbf{s}}\left( l \right)$ chosen as a line that intersects at and is perpendicular to the center of the surface current support, i.e. ${\mathbf{s}}\left( l \right) = {{\mathbf{r}}_{\mathbf{c}}} + l{\widehat {\mathbf{n}}_{\mathbf{c}}}$, ${\widehat {\mathbf{n}}_{\mathbf{c}}}$ is the head unit normal pointing inwards at the point nearest to the coil center. In accordance, the stimulation depth is
  \begin{equation*}\begin{array}{*{20}{l}}d_M(\lambda)= \displaystyle\max_{l \ge 0}\left\|\mathbf{s}(C) - \mathbf{s}(l)\right\|\\[6pt]\text{subject to:}\\[4pt]\left\{\begin{array}{l}\mathbf{E}\!\left(\mathbf{s}(l)\right)\cdot\widehat{\mathbf{t}}\;\ge\; E_{\mathrm{TARG}}\\[4pt]\mathbf{s}(l)\in\mathrm{Brain}\end{array}\right.\end{array}\end{equation*}
  where ${\mathbf{s}}\left( C \right)$ denotes the point on the cortex closest to ${{\mathbf{r}}_{\mathbf{c}}}$. For example, figure 1(B) depicts the coil placed centered about and oriented perpendicular to the *z*-axis. In this case, the line in blue denotes ${\mathbf{s}}\left( l \right)$ = $\left( {90 - l} \right){\text{ }}\widehat {\mathbf{z}}{\text{ }}mm$. Furthermore, markers were included at ${\mathbf{s}}\left( C \right) = - 20{\text{ }}\widehat {\mathbf{z}}{\text{ }}mm$ and the lowest point with E-field above threshold ${\mathbf{s}}\left( {C + {d_M}\left( \lambda \right)} \right)$. The value of ${d_M}\left( \lambda \right)$ is the distance between these two points and the choice of ${\widehat {\mathbf{n}}_{\mathbf{c}}}$ pointing toward the brain results in ${\mathbf{s}}\left( {C + {d_M}\left( \lambda \right)} \right)$ being the deepest point stimulated.
(iii) *Minimum energy:* TMS pulses have relatively low-frequency temporal variation and their induced magnetic field is negligibly affected by the presence of the head. The magnetic energy stored in the current distribution can be computed using the Biot–Savart law [35] as
  \begin{equation*}W\left( {{\lambda }} \right) = \frac{{{{{\mu }}_0}}}{{8{{\pi }}}}\mathop \int \limits_{{\Omega }}^{} {\mathbf{I}}\left( {{\mathbf{r}};{{\lambda }}} \right) \cdot \mathop \int \limits_{{\Omega }}^{} \frac{{{\mathbf{I}}\left( {{\mathbf{r}}^{\prime};{{\lambda }}} \right)}}{{\left\| {{\mathbf{r}} - {\mathbf{r}}^{\prime}} \right\|}}{{\mathrm{d}}^{\mathrm{3}}}\mathbf{r}^\prime{{\mathrm{d}}^{\mathrm{3}}}\mathbf{r},\end{equation*}

where ${\mu _0}$ is the permeability of free space.

In addition to the aforementioned figures of merit, we combined $V$ and $d$ to define spread ($S)$ as the average transverse surface area of the stimulated region, calculated as $S = V/{d_M}$ [21, 36].^8^8Directional full-width-at-half-maximum metrics summarize the stimulated region using two principal axes. Here we use ${S_{1/\alpha }}$ (average transverse area of the suprathreshold region) because many designs produce non-elliptical stimulation footprints [21], and a single full-width-at-half-maximum ellipse can underestimate off-axis stimulation. Reducing either $V$ or $S$ is equivalent to an increase in focality. Moreover, safety considerations impose limits on the maximum E-field strength. For a given $\alpha $, we assumed that E-field strength exceeding $\alpha {E_{{\mathrm{TARG}}}}$ in the brain is unacceptable. Therefore, currents in the span of the modes that result in an E-field that exceeds $\alpha {E_{{\mathrm{TARG}}}}$ in the brain were excluded from the admissible designs. Throughout, we used thresholded spread metrics, ${S_{1/\alpha }}$, where ${\text{ }}\alpha &gt; 1$. In prior research, a common selection was $\alpha = 2$. In this scenario, $V$, ${d_M}$, and $S$ are equal to the figures of merit ${V_{1/2}}$, ${d_{1/2}}$, and ${S_{1/2}} = {V_{1/2}}/{d_{1/2}}$, respectively [20, 21, 36, 37]. Consequently, this led to $V$ representing the sub-volume of the brain where the E-field equals or exceeds half of its peak value, and ${d_M}$ representing the greatest depth where the E-field equals or exceeds ½ of its peak value.

In many instances the peak E-field on the cortex was less than twice ${E_{{\mathrm{TARG}}}}$. Furthermore, TMS pulse current could be increased or decreased, thereby allowing for the coil to stimulate deeper or shallower regions. As such, the energy and spread of a fixed coil is not a constant but a function of targeted depth. To account for this in the coil benchmarks we additionally considered the stimulation volume, spread, and energy for each coil as a function of targeted depth, i.e. ${V_d}\left( d \right)$, ${S_d}\left( d \right)$, and ${W_d}\left( d \right)$, respectively, where $d \in (0,{d_M}]$. ${V_d}\left( d \right)$ and ${S_d}\left( d \right)$, and ${W_d}\left( d \right)$ were computed by driving the coil with a current that results in an E-field of ${E_{{\mathrm{TARG}}}}$ at $d$. Furthermore, targeted depths that result in a peak cortical E-field above $\alpha {E_{{\mathrm{TARG}}}}$ were considered unreachable and excluded from the evaluation of ${V_d}\left( d \right)$, ${S_d}\left( d \right)$, and ${W_d}\left( d \right)$.

Finally, we considered the design of coils with the alternative choice of $\alpha = \sqrt 2 $ used in some publications [11]. We found that ${V_d}\left( d \right)$, ${S_d}\left( d \right)$, and ${W_d}\left( d \right)$ for designs with either $\alpha = 2$ or $\alpha = \sqrt 2 $ achieve similar trade-offs for shallow depths, and the ones designed with $\alpha = 2$ achieved superior performance for larger depths. As such, we used and recommend $\alpha = 2$ to design fdTMS coils.

### 2.3. Coil winding generation

Here we discuss how we converted the current density ${\mathbf{I}}\left( {{\mathbf{r}},\lambda } \right) = \mathop \sum \nolimits_{i = 1}^{{n_p}} {\lambda _i}{{\mathbf{J}}_i}\left( {\mathbf{r}} \right)$ into coil windings. Our approach started by adopting the standard coil winding generation approach that defines the stream function of the current ${\mathrm{Sr}}\left( {{\mathbf{r}},\lambda } \right) = \mathop \sum \nolimits_{i = 1}^{{n_p}} {\lambda _i}{N_i}\left( {\mathbf{r}} \right)$, where ${\mathbf{I}}\left( {{\mathbf{r}},\lambda } \right) = - \widehat {\mathbf{n}} \times \nabla {\mathrm{Sr}}\left( {{\mathbf{r}},\lambda } \right)$. The contour lines of the stream function point in the direction of the surface current and trace out regions of constant current. Furthermore, since the current is dependent on the gradient of the stream function, an approximation of the surface current was made by placing wires on contour lines that are an equal elevation distance apart (i.e. equispaced contour intervals). For example, if we choose $M$ equispaced contour intervals, then, the wires are placed at contour elevation levels of

\begin{align*}\begin{array}{*{20}{c}} {{\mathrm{ele}}{{\mathrm{v}}_i} = \min \left( {{\mathrm{Sr}}\left( {\mathbf{r},\lambda } \right)} \right) + \left( {i - \frac{1}{2}} \right){{{\Delta }}_{{\mathrm{sr}}}}} \\ {{{{\Delta }}_{{\mathrm{sr}}}}\left( M \right) = \frac{1}{M}\left( {{\mathrm{max}}\left( {{\mathrm{Sr}}\left( {{\mathbf{r}},\lambda } \right)} \right) - \min \left( {{\mathrm{Sr}}\left( {\mathbf{r},\lambda } \right)} \right)} \right)} \end{array}\end{align*}

where $i = 1, \ldots ,{\text{ }}M.$ These wires were then typically connected serially to generate a coil design. This approach has been shown to yield coil designs that match the surface current distribution and by proxy E-fields. For a fixed capacitor capacitance and voltage, the peak current and resonant frequency are inversely proportional to the square root of the coil inductance. The value of $M$ is typically chosen to result in a coil design that matches a desired inductance that results in a specified TMS pulse width and peak current.

For fdTMS coil designs, the contour lines typically concentrate near the coil center, and it is difficult to attain the TMS driver required inductance ($ \ge 8.5{ }\,\mu {\mathrm{H}}$) without using multiple layers of wire. Adding multiple winding layers results in a design that is less energy efficient than a single layer design. To achieve the desired inductance while accommodating enough contour intervals to achieve a desired inductance we adopted two strategies.

One evaluated strategy was to add constraints to the optimization to ensure that the stream function would allow enough concentric loops to fit the required number of turns of the windings. For a given choice of $M$, the closest any two windings can theoretically be is $dis{t_{min}} = {{{\Delta }}_{sr}}\left( M \right)\max \left\| {{\mathbf{I}}\left( {{\mathbf{r}},{{\lambda }}} \right)} \right\|$. Following established practice [38], we set the additional constraints to our fdTMS design framework to guarantee that ${\mathrm{dis}}{{\mathrm{t}}_{{\mathrm{min}}}} \ge {W_{{\mathrm{wire}}}}$, where ${W_{{\mathrm{wire}}}}$ is the wire width. This was done by enforcing ${{{\Delta }}_{sr}}\left( M \right)\max \left\| {{\mathbf{I}}\left( {{\mathbf{r}},{{\lambda }}} \right)} \right\| \le {W_{wire}}$, i.e. by constraining the maximum value of the *L*-infinity norm and as such ensuring fit for wires of width ${W_{{\mathrm{wire}}}}$. Note that this approach differs from previous optimizations that used the *L*-infinity norm as the cost function, which have been shown to yield designs with more spread-out concentric windings [24], or from alternative winding-spacing optimization approaches [39, 40] since our results only needed to satisfy a fixed constraint to fit a prescribed wire size. Correspondingly, outside this region, the optimization is unconstrained and may generate currents where it best supports deep focal stimulation.

We iteratively implemented this constraint by first running the fdTMS framework and adding constraints where the condition was violated. The value of ${{{\Delta }}_{{\mathrm{sr}}}}\left( M \right)$ was determined using the stream function of the previous design iteration. The constraint of $\max \left\| {{\mathbf{I}}\left( {{\mathbf{r}},{{\lambda }}} \right)} \right\|$ was approximated by 16 linear constraints by using the same approach that was used for the E-field constraint in [11]. This procedure converged after 2–5 iterations for all cases tested here. For any given $M$, ${\mathrm{dis}}{{\mathrm{t}}_{{\mathrm{min}}}}$ could only be reduced marginally while maintaining the same performance. As a result, these additional constraints still required the use of multiple layers.

The second evaluated strategy adopted a hybrid approach where the coil has multiple layers only on critical regions where the concentric windings are densest. This strategy exploited the fact that the induced E-field is relatively insensitive to small perturbations of the winding layout. Instead of using uniformly spaced stream-function contours, we selected non-uniform contour levels as winding locations and chose them to minimize the mismatch between the ideal fdTMS E-field and the E-field produced by the hybrid-layer coil implementation. Specifically, the majority of fdTMS coils consisted of three distinct types of sub-coils: figure-8, biasing, and cancellation windings. The biasing and cancellation windings allowed enough space to be implemented using one layer, while the figure-8 winding required three layers. We first partitioned the coil surface ${{\Omega }}$ into the three subregions containing the figure-8, biasing, and cancellation loops, respectively. For each part, we specified the number of concentric loops in our implementation and number of layers. Each concentric loop consisted of wire placed at a contour line. As a result, the design was specified by the contour line elevation levels and layers for each coil sub-region. The contour line height levels were determined by running an optimization to minimize the error between E-fields generated by the coil and the optimal surface current.

To numerically achieve the above objective, the E-fields of the coil and the optimal surface current were sampled uniformly at 46 532 points on a spherical shell and assembled into $46\,532 \times 3$ matrices ${{\mathbf{E}}_{{\mathbf{Coil}}}}$ and ${{\mathbf{E}}_{{\mathbf{Surf}}}}$, respectively. Furthermore, to regularize the optimization, we added an energy penalty equal to ${10^{ - 5}}{\|{\mathbf{E}}_{{\mathbf{ideal}}}\|}_F{{\mathrm{W}}_{{\mathrm{coil}}}}$, where ${\|{\mathbf{E}}_{{\mathbf{ideal}}}\|}_F$ is the Frobenius norm of ${{\mathbf{E}}_{{\mathbf{ideal}}}}$ and ${W_{{\mathrm{coil}}}}$ is the energy required by the coil to generate an E-field that optimally matches ${{\mathbf{E}}_{{\mathbf{ideal}}}}$. Finally, a constraint was added to ensure that only designs with concentric loops 2.2 mm apart are admissible. The final optimization was done using MATLAB’s fmincon to minimize the function ${\left\|{\mathbf{E}}_{\mathbf{coil}} - {\mathbf{E}}_{{\mathbf{ideal}}} \right\|_F} + {10^{ - 5}}{\left\| {{{\mathbf{E}}_{{\mathbf{ideal}}}}} \right\|_F}{{\mathrm{W}}_{{\mathrm{coil}}}}$. Note that the optimal continuous surface-current solution was obtained using the fdTMS framework, and fmincon was only used to approximate it by coil windings.

The approaches described above resulted in designs consisting of disconnected loops. The loops were manually connected serially. The connections were chosen with the following considerations in mind: branching from concentric windings should be done far from the coil center, branching between coils was both chosen where neighboring coils are closest, and the curvature of the transitions between coils should be small enough to enable practical winding of the coils.

To benchmark the procedure described above, we generated winding patterns for fdTMS coils that match or exceed the penetration depth of existing TMS coils. Specifically, the above procedure was applied to design fdTMS coils with ${d_{1/2}}$ ⩾ 1.01, 1.31, and 1.57 cm, which are the ${d_{1/2}}$ values of MagVenture B35, B65 and B80 coils, respectively. In each case, we chose the number of turns to result in an inductance $ \ge 8.8{ }\,\mu {\mathrm{H}}$.

### 2.4. Coil shape and former design

**Figure 2.**
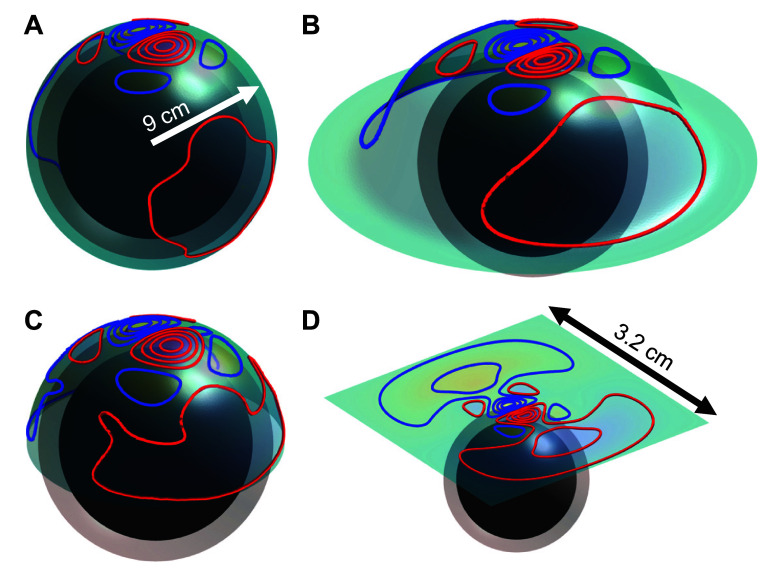
Evaluated fdTMS coil supports (surface shapes): (A) sphere, (B) hat, (C) half-sphere and (D) square support placed over the spherical head model. The inner-most sphere is the brain region.

The hat-shaped coil support was derived from five MRI-based head models [41] using SimNIBS [42]. Specifically, we densely sampled coil placements on the motor strip of each subject. Then, the most conformal radially symmetric shape that would allow orienting and placing the coil on all the candidate motor strip positions was chosen. The general shape is given in figure 2(B). To compare the coil shape’s effect on performance, we additionally considered sphere (figure 2(A)), half-sphere (figure 2(C)), and square (figure 2(D)) coil supports.

**Figure 3.**
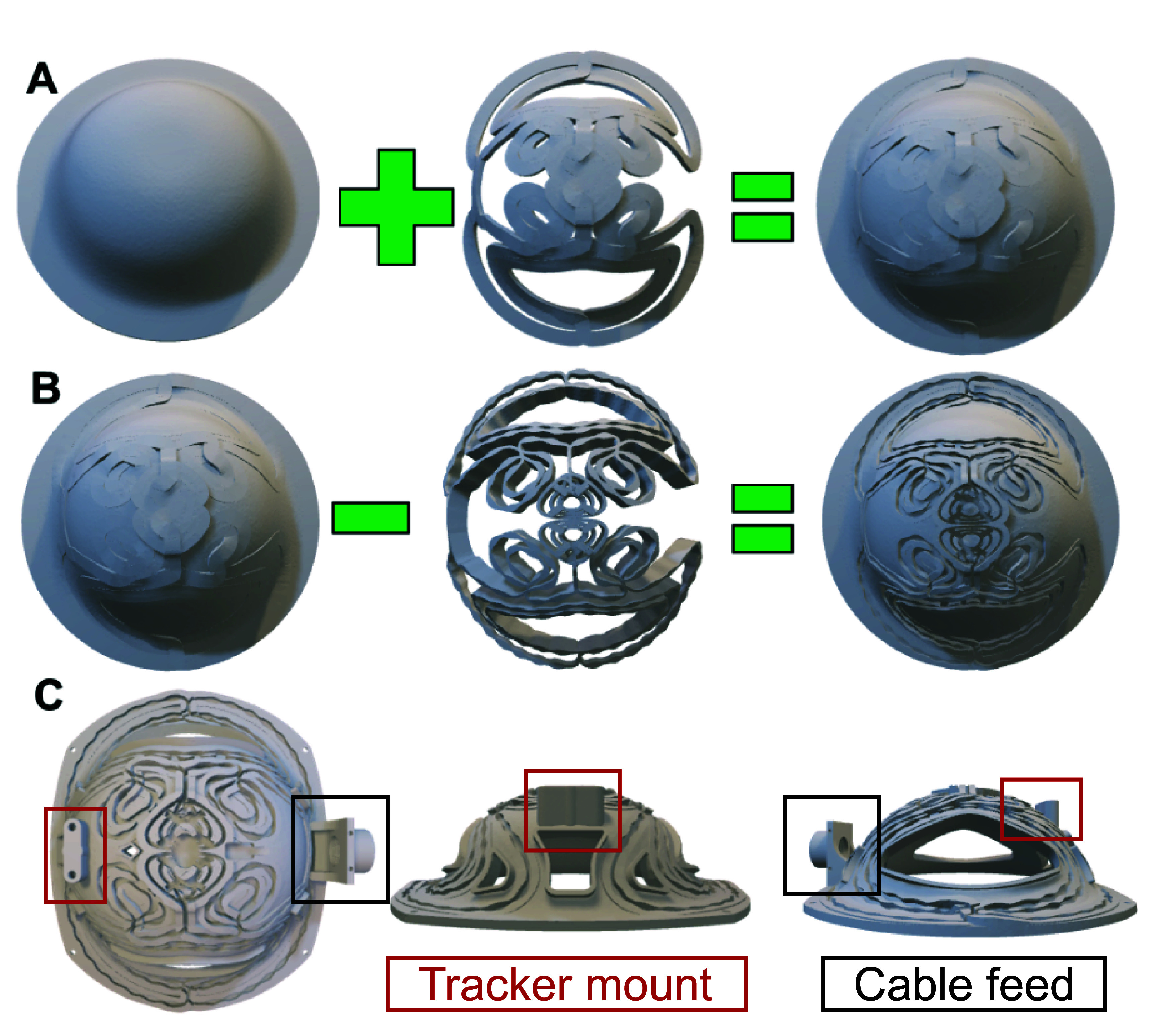
Fabrication steps of fdTMS coil hat-shaped former. (A) Coil support mesh is merged with a thick and tall wire mesh. (B) Ribbed wire mesh is subtracted from the merged mesh in (A). (C) Three views of the final mesh with additional mounting supports for cable feed and neuronavigation tracker, and holes cut out for ventilation and head visibility.

We generated several meshes by extruding the coil about its normal direction and converting the filamentary wire representations to thick wire meshes (figure 3). First, a thin version of the coil support and thick and tall winding meshes were merged (figure 3(A)). This first step allowed us to have tall enough coil channel grooves without requiring a heavy coil support. Second, ribbed windings were subtracted from the merged mesh (figure 3(B)). The resulting mesh has grooves where the windings will reside. The narrower segments of the grooves mechanically hold the wire in place, whereas the wider stretches of the grooves reduce friction during the wire insertion and provide space to inject epoxy to bond the wire to the former.

### 2.5. Coil fabrication

We built two of the fdTMS coils—one to target a depth of 1.31 and the other to target a depth of 1.57 cm to match the depth characteristics of the Cool-B65 and D-B80 coils, respectively (MagVenture A/S). The coil former (figure 3(C)) was 3D printed using selective laser sintering of Nylon PA 12. The top and bottom surfaces of the former were then sprayed with electrical sealant to provide additional insulation. A 12 AWG (2.05 mm diameter) round magnet wire was wound in the former grooves following the winding path. The wire diameter was constrained by the geometry and number of turns of the computationally optimized windings. The wire diameter is compatible with the copper skin depth of 0.92–2.06 mm in the dominant frequency spectrum of TMS pulses (1–5 kHz) [43, 44]. During the winding process, small drops of hot glue were used intermittently throughout the path to secure the wire in place. Once the entire coil had been wound, quick-set epoxy was injected into the grooves and allowed to fully cure for 24 h. Finally, the two magnet wires exiting the coil were soldered to copper connectors that are then attached to a cable assembly compatible with MagPro TMS devices (MagVenture A/S). To monitor for safe coil operation, a temperature sensor wired to the cable assembly was affixed to the former near the center of the coil winding.

### 2.6. Coil electrical and safety testing

The coil windings and cable conductors were tested at 10.7 kV applied for 60 s relative to the external coil surface to ensure safe electrical insulation per standard IEC 60601-1 [45]. The coil integrity was also validated by delivering single TMS pulses up to the maximum output of 1800 V and 600 J in power pulse mode of the TMS device (MagPro X100 including MagOption) [46]. The coil inductance and resistance were measured at 1 kHz with an LCR/ESR meter (B&K Precision, model 889A).

### 2.7. Coil E-field measurement

**Figure 4.**
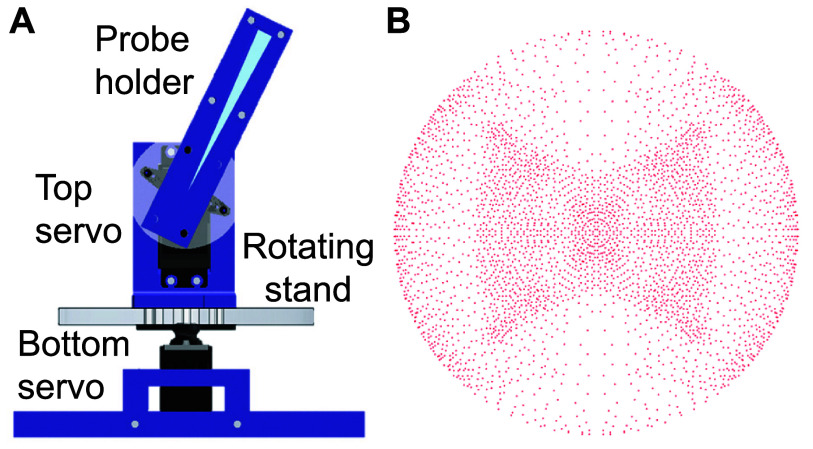
Robotic E-field measurement rig. (A) The measurement probes are attached with their apex centered at the axis of rotation of bottom and top servo motors and span a hemisphere. (B) Top view of the 3620 probe measurement positions.

To validate the design of the fdTMS coils, their E-field as well as the E-field of conventional figure-8 coils (MagVenture Cool-B65 and D-B80) were measured across a hemispherical shell using isosceles triangle probe coils mounted on a robotic rig [47]. The triangle probe sits in air and does not necessitate saline or other conductive media, since its structure allows the probe to measure the total E-field induced magnetically in a spherical volume conductor [47]. The robot consists of a horizontal stand rotated by a ‘bottom’ servo motor controlled by an Arduino board. The horizontal stand holds another ‘top’ servo actuating an E-field probe incorporating four isosceles triangle sensing coils. Two of the triangle coils have a height of 60 mm and base width of 4 mm, and the other two have a height of 70 mm and base width of 5 mm. The equal height sensing coils were placed orthogonal to one another, and all the probes were placed with their apex centered at the axis of rotation of both servo motors as shown in figure 4(A).

The measured TMS coil was centered directly above the apex of the probe (i.e. above the axis of rotation of the two servo motors). The TMS coil surface was positioned 8.7 cm above the apex of the probe, corresponding to 1.7 cm from the approximate brain surface. The probe was programmed to measure the E-field at a total of 3620 distinct positions as shown in figure 4(B) using an Arduino and MATLAB code. The measurement positions were more densely concentrated along the top of the sphere where E-field variations are sharper. The measurement positions are at most 1° apart. These measurements were then used to generate vector maps of the E-field at the cortical surface of a spherical brain model (7 cm shell) and 1 cm below (6 cm shell) [47]. (Note: the probe measures the average E-field along its base. Because of its finite width, each reported value corresponds to the average E-field over this width, centered on the reported location.)

During the E-field measurements, TMS pulses were delivered to the coil using a MagPro R30 device (MagVenture A/S) at 27% of maximum stimulator output (MSO). The stimulator was configured to send a sequence of biphasic pulses at a rate of 0.5 pulses per second in 10 trains of 364 pulses each with an intertrain interval of 1 s.

### 2.8. Head models

A common spherical head model consisting of a homogenous sphere with radius of 8.5 cm was used for the fdTMS coil design and evaluation [11, 21]. The head model consisted of two concentric spheres each centered about the origin and having radii of 7.0 cm and 8.5 cm, respectively. The inner sphere corresponds to the brain, and the outer shell—to the cerebrospinal fluid, skull, and skin. Since TMS does not induce any radial currents in a spherical conductor and the E-field is independent of the specific conductivity according to the quasi-static modeling approximation, a single conductivity of 0.33 S m^−1^ was used for the whole sphere [48]. For each simulation (figures 2(A)–(D)), the center of the coil in Cartesian coordinates was ${{\mathbf{r}}_{\mathbf{c}}} = \left( {0,0,0.09} \right)$ and ${\widehat {\mathbf{n}}_{\mathbf{c}}} = - \widehat {\mathbf{z}}$. Correspondingly, depth was measured along the $ - z$ direction starting from $z = 7.0$ cm and the coil E-field at the target was designed to be *y-*oriented (i.e. $\widehat {\mathbf{t}} = \widehat {\mathbf{y}}$). We chose ${\mathbf{t}} = \widehat {\mathbf{y}}$ without loss of generality: in the spherical model, $\widehat {\mathbf{x}}$ is equivalent up to rotation, and $\widehat {\mathbf{z}}$ corresponds to the radial direction for this placement, along which the coil does not produce any field [20]. Analytical expressions for the E-field generated inside the spherical head model are given in [11] and used to determine the E-field generated by the surface currents in the context of the fdTMS coil design optimization.

### 2.9. Coil performance evaluation with finite element simulation

**Figure 5.**
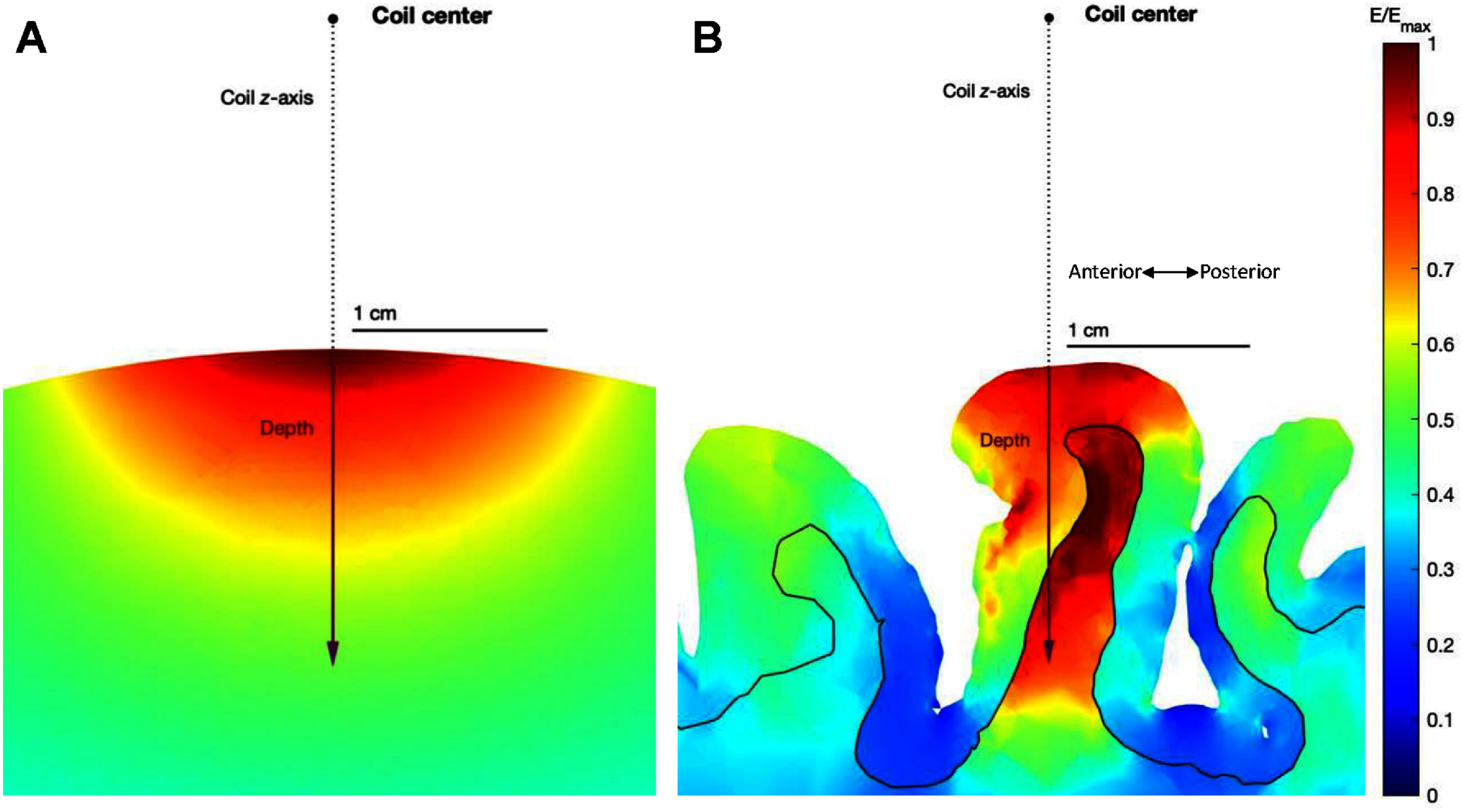
Coil position and orientation relative to the ‘brain’ in the (A) spherical head model and the (B) motor cortex hand knob in the precentral gyrus of a realistic head mo the boundary between the gray matter and white matter is contoured in black.

Using the same definitions for stimulation volume *V*, energy *W*, and depth of stimulation *d*, we characterized the stimulation volume and energy of fdTMS and, for comparison, standard figure-8 coil models over a range of target depths (figure 5) using finite element simulations in SimNIBS [42]. The spherical head model was recreated in SimNIBS with 30.7 million tetrahedra. The fdTMS coil definitions were imported into SimNIBS by generating a voxel grid of primary E-field samples and storing them as NIfTI files, and the native SimNIBS models for three conventional TMS coils were used. The E-field strengths on the outer (scalp) and inner (cortical) surfaces of the spherical model were calculated at the center of each triangle that constitutes the corresponding surface mesh during finite element analysis. We then calculated the peak E-field strength on the cortical surface over a range of depths and characterized the distribution of the E-field on the model scalp surface relative to the target E-field to compare coil tolerability.

We also evaluated coil focality in the realistic *Ernie* head model from SimNIBS 4.1 [42] by centering each TMS coil over the hand knob region of the motor cortex at the same location and orientation to approximate coil placement during motor thresholding, with the same distance between the center of the coil and the scalp as in the spherical head model. *Ernie* consists of white matter (0.126 S m^−1^), gray matter (0.275 S m^−1^), cerebrospinal fluid (1.654 S m^−1^), blood (0.6 S m^−1^), compact bone (0.008 S m^−1^), spongy bone (0.025 S m^−1^), eye balls (0.5 S m^−1^), muscle (0.16 S m^−1^), and scalp (0.465 S m^−1^) compartments. Depth was measured along the *z*-axis of the coil in accordance with the coil axes orientations in SimNIBS, with *d* = 0 defined at the intersection with the gray matter surface (figure 5). Since the neural activation by TMS is superficial [49], the focality metrics were computed only in the gray matter.

### 2.10. Coil performance evaluation in human subjects

*Participants:* this study was approved by the Duke University Health System Institutional Review Board (Pro00107556). Healthy participants were recruited, completed informed consent, and were compensated $20 h for their time. All procedures were conducted on the same day, unless time constraints necessitated the subjects to return to complete the session.

*MRI and neuronavigation:* anatomical MRI T1-weighted images were acquired and used in Brainsight (Rogue Research, Montreal, Canada) for TMS neuronavigation (parameters reported previously [50]). For one subject an MRI could not be acquired due to permanent makeup with unknown MRI compatibility, and the MNI head template was used instead.

*Electromyography recordings:* electromyographic (EMG) recording of motor-evoked potnetials (MEPs) in three muscles of the dominant right hand—abductor pollicis brevis (APB), first dorsal interosseous (FDI), and abductor digiti minimi (ADM)—was conducted with Ag/AgCl foam electrodes (Kendall 133, Covidien LLC) and an EMG amplifier (BrainAmp ExG, BrainVision, USA). EMG of the biceps and deltoid was collected as well, but was not included in the final analysis since there was not substantial baseline activation in these muscles. The amplifier bandpass filtered the EMG signal between 0.1 Hz and 1000 Hz and sampled MEPs with 16-bit resolution at 5 kHz. Recordings that showed activity of more than 40 *μ*V peak-to-peak amplitude within the 200 ms interval immediately before the TMS pulse were marked as facilitated and excluded from the analysis. The MEP amplitude was defined as the difference between the maximum and minimum values of the processed EMG waveform occurring 20–50 ms after the TMS stimulus for hand muscles.

*TMS coils and pulse type:* for all coils and pulse types, a MagPro X100 with MagOption (MagVenture, Farum, Denmark) pulse generator was used. Two commercial MagVenture coils were used in the comparison: Cool-B35 HO (B35) and Cool-B65 (B65). The MagVenture D-B80 (B80) was included in initial testing, but its angled design precluded reliable placement over the hand-knob region, and therefore this coil was excluded from the experimental study. Two prototype fdTMS coils (F65 and F80) were included in the study; they were designed to match or exceed the stimulation depth of the B65 and B80 coils, respectively. The design for the F35 coil, based on the depth of the B35, was not implemented, since its energy requirements well exceeded those of conventional pulses. For all coils, the MagPro stimulator was set to produce monophasic TMS pulses in power mode (to lower the resting motor threshold (RMT)) with the current direction reversed (corresponding to the conventional posterior–anterior induced initial current direction in primary motor cortex). As is standard during TMS procedures, the study subjects and TMS operators wore ear plugs for hearing protection [51].

At the start of the study, the MagVenture stimulator software did not permit the generation of monophasic pulses in power mode with the B35 coil. Therefore, for the first three completed subjects, biphasic mode was used with standard current direction (posterior–anterior direction of the second, dominant phase of the induced current). After a software update from MagVenture, power mode monophasic pulses were enabled for the B35 coil and were used in the remaining six subjects. Since for either pulse shape the dominant current direction was the same and the pulse intensity was delivered relative to the pulse-specific RMT, the pulse shape was not expected to have a significant effect on the results. When comparing the coil RMTs, the statistical analysis was conducted both with and without the biphasic RMTs, and the results were not affected.

*RMT determination:* for each coil, the hotspot producing the strongest activation of the FDI was identified with a systematic search over the primary motor cortex hand knob area with the coil oriented at 45° relative to the midline [50]. RMTs defined as the TMS intensity producing an average MEP of 50 *μ*V peak-to-peak with the muscle relaxed, was then titrated with the MTAT 2.0 program [52], and convergence was confirmed by the administration of additional pulses during which the RMT estimated by MTAT did not vary by more than 1% of MSO.

**Figure 6.**
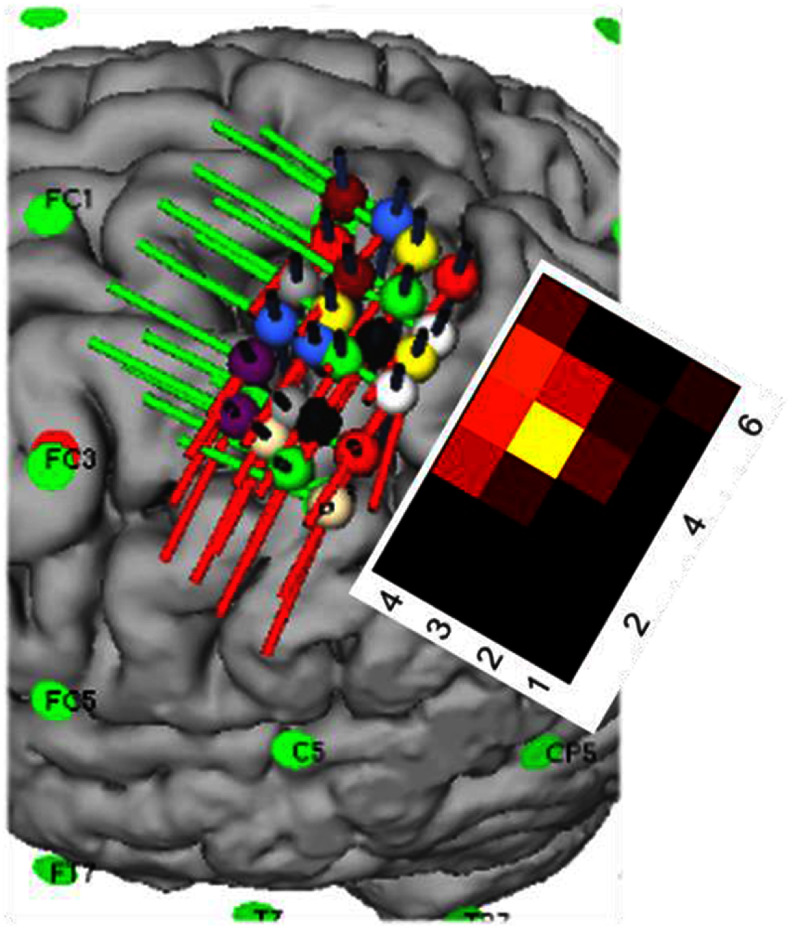
Example of motor cortex sampling maps generated in Brainsight and the grid output plot (inset).

*Motor area mapping:* to evaluate the extent of recruitment of the cortical muscle representations, each coil was placed sequentially on a grid of 6 × 4 scalp locations designed to map the response of the motor cortex hand knob. The grid was warped to the cortical surface reconstructed from the MRI data in Brainsight. The angular orientation of the coil and grid was set to maintain the coil handle at 45° from the posterior side of the longitudinal fissure (−135° in the Brainsight system). This resulted in the length of the individual grids generally aligning to the central sulcus. The grid point spacing was approximately 5 mm on the cortex, and the grid approximately covered the latero–medial extent of the hand knob motor area (figure 6).

MEPs were recorded from the three hand muscles (FDI, APB, ADM) simultaneously. Five non-facilitated MEP samples at 105% RMT for the respective coil were acquired from each grid point. For a sample to be included, there had to be no facilitation/pre-pulse muscle activity in any of the three recorded muscles. Initially, we explored conducting the mapping at 110% RMT, but this was less likely to be tolerable with the fdTMS coils.

For consistent grid location across coils, the grid for each participant was centered on their Brainsight estimated scalp location corresponding to the cortical FDI hotspot for the B65 coil. Therefore, the FDI hotspot and RMT were determined first with the B65 coil. Subsequently, the FDI hotspot and RMT for the remaining coils with the motor mapping for all coils were performed in a pseudorandomized counterbalanced order.

*MEP preprocessing:* all included MEP samples underwent TMS artifact removal: Exponentials were fitted to the EMG trace after the TMS pulse, and this baseline shift was subtracted. The post-MEP signal average (50–250 ms) was subtracted and signals were then high-pass filtered with an acausal approach to minimize TMS artifact spread in the MEP time window [53, 54]. Acausal filtering was implemented by reversing the signal trace in time and high-pass filtering it (4th-order Butterworth, cutoff frequency of 40 Hz). To minimize EMG signal noise in the MEP map visualizations, the traces of the five MEP samples for each grid point within subject and muscle were averaged before the peak-to-peak amplitude was measured, and the amplitudes were plotted on a log scale.

*Coil experience:* after the map for each coil was acquired, subjects were asked to rate their experience of each coil in various terms of discomfort and location of discomfort. Participants were asked to rate the annoyance, pain, and muscle twitches caused by each coil on a scale from zero to ten, with zero being not severe at all and ten being the most severe. Participants also reported the location of pain and muscle twitches in these evaluations. At the end of the session, participants completed a routine TMS side effects questionnaire.

*Statistical analysis:* statistical analyses were carried out with JMP Pro 17.2.0 (JMP Statistical Discovery LLC, USA). The RMT, MEP amplitude, and sensation ratings data (annoyance, pain, and muscle twitching) were analyzed with mixed effects models with coil as a fixed effect and subject as a random effect. Significant (*p* < 0.05) results were followed up with a Tukey HSD test to compare individual coils.

Using the data from the RMT titration, the FDI MEP amplitudes were first compared across coils at 100% FDI RMT to check for proper matching of the stimulation strengths. The MEP amplitudes were log-transformed to improve the normality and homoscedasticity of their distributions [55–57].

The focality of the coils for recruiting each muscle was assessed by comparing the number of MEPs that exceeded 100 *μ*V peak-to-peak [58] during the grid-based mapping at 105% FDI RMT. The binarized MEP responses were analyzed with a generalized linear mixed effects model with Tukey HSD post-hoc comparisons with coil, muscle, and their interaction as fixed effects and subject as a random effect.

## 3. Results

### 3.1. Energy vs. focality for various coil supports

**Figure 7.**
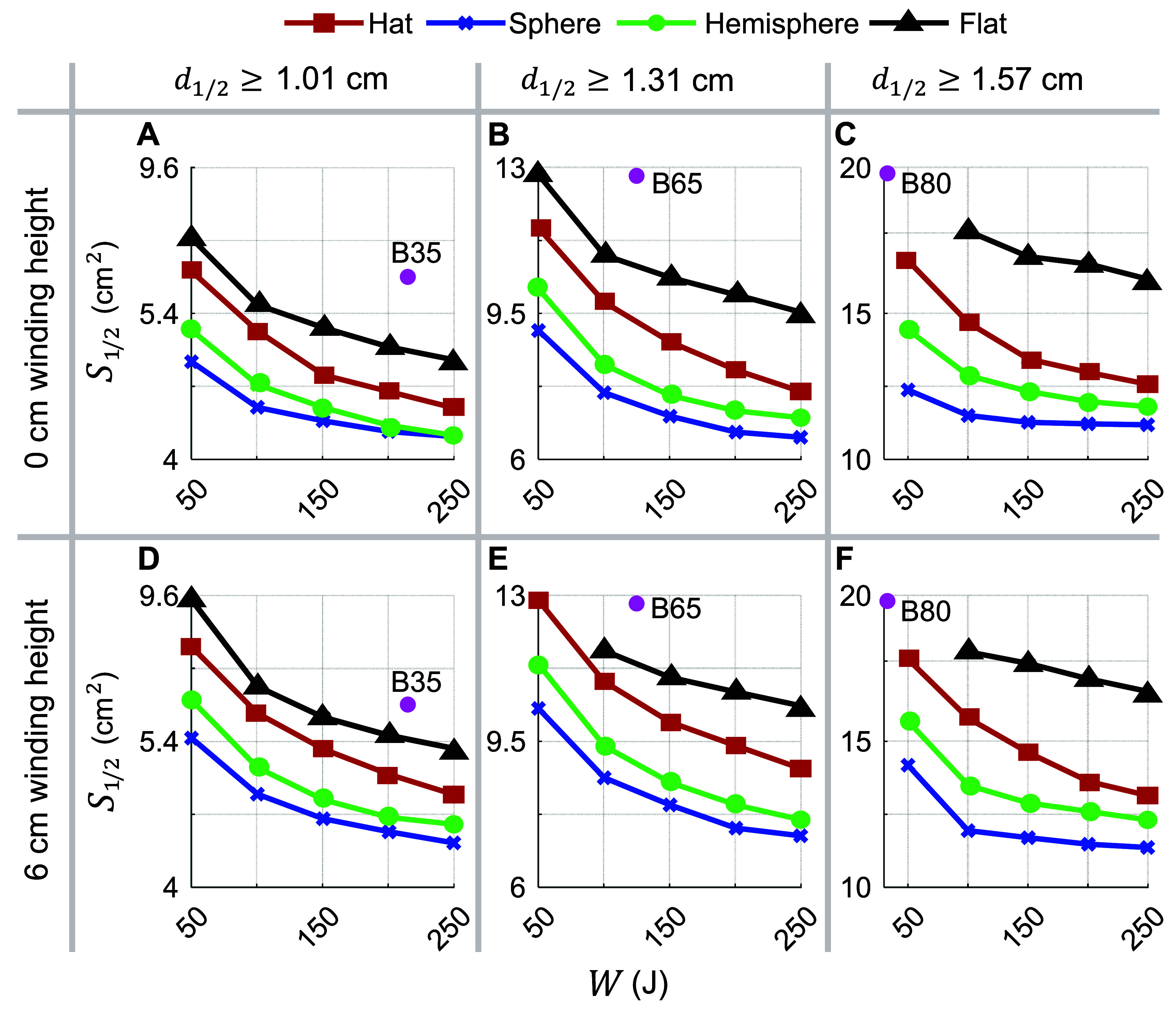
Numerically computed trade-off between E-field spread above $1/2$ of peak value and coil energy for fdTMS coils with different support surfaces (see legend) and (A)–(C) filamentary winding with 0 mm height or (D)–(F) winding height of 6 mm. The columns correspond to coil designs with target depths of (A), (D) ${d_{1/2}} \ge 1.01$ cm; (B), (E) ${d_{1/2}} \ge 1.31$ cm; and (C), (F) ${d_{1/2}} \ge 1.57$ cm, matching commercial coils MagVenture B35, B65, and B80, respectively, whose performance is denoted with pink dots for comparison. In this comparison, windings of each coil are not connected and are treated as separate closed loops.

Here we analyze trade-offs between focality and required energy for various target depths and coil topologies. Specifically, we compared the energy vs. focality of the hat coil relative to the sphere, hemisphere, and flat coils of our previous work [20] and of our current work including wire thickness. Figure 7 shows energy versus spread curves for target depths ${d_{1/2}} \ge \left\{ {1.01,{\text{ }}1.31,{\text{ }}1.57} \right\}$ cm, respectively corresponding to the B35, B65, and B80 coils. Coils with spherical support were the most conformal and they are more focal than the others for matched depth and energy. The coils with hat support exhibited performance that is better than the square support coils and worse than the hemispherical coils. The efficacy of conformal relative to non-conformal coils increased with depth: The flat coils preformed nearly as well as the hat shaped ones for ${d_{1/2}} \ge 1.01$ cm (figures 7(A) and (D)), whereas flat coils were significantly inferior for ${d_{1/2}} \ge 1.57$ cm (figures 7(C) and (F)). Independent of depth, the fdTMS coils outperformed the conventional coils by either energy for matched spread or spread for matched energy, consistent with our prior findings [20].

**Figure 8.**
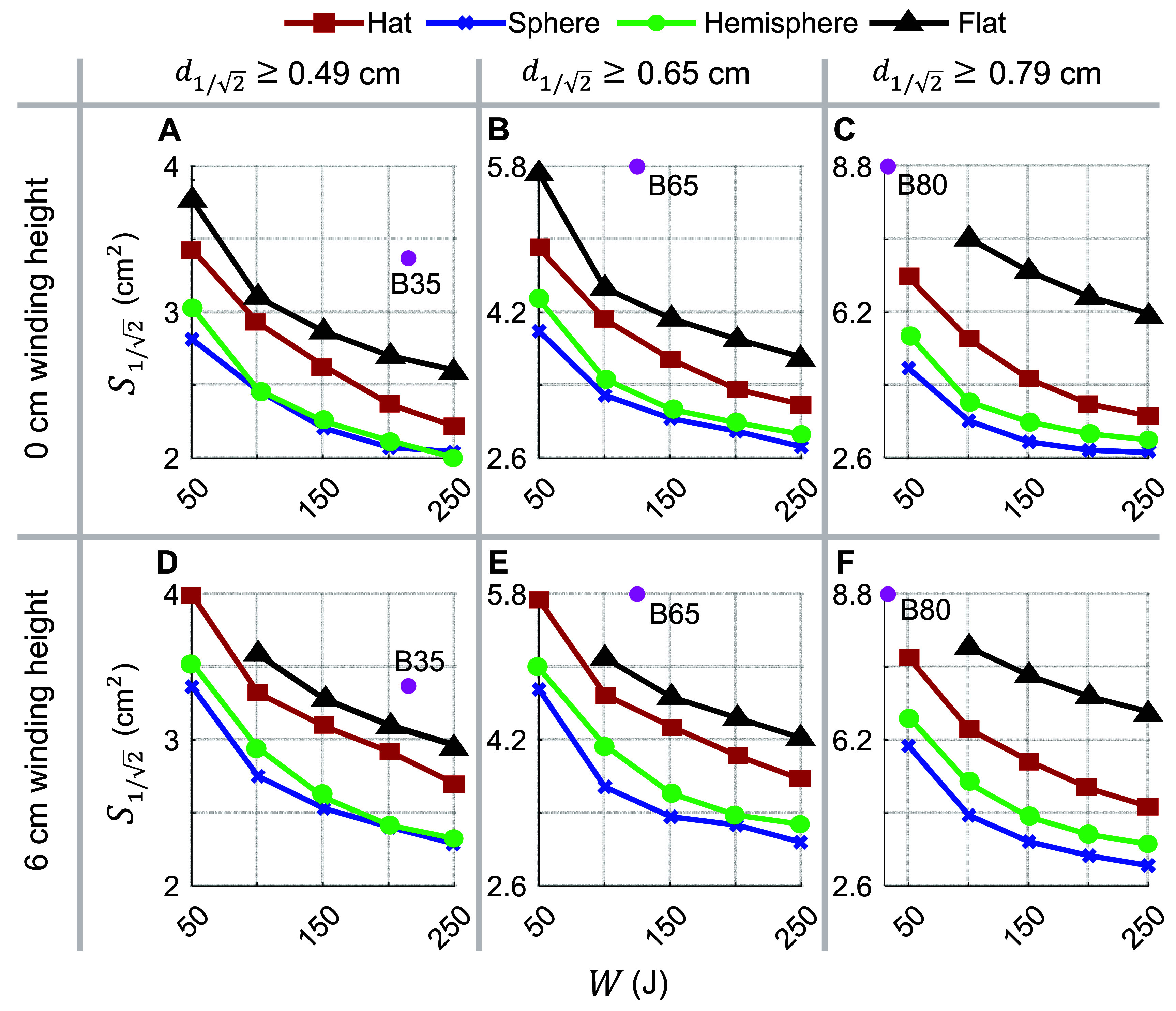
Numerically computed trade-off between E-field spread above $1/\sqrt 2 $ of peak value (alternative metric) and coil energy for fdTMS coils with different support surfaces (see legend) and (A)–(C) filamentary winding height or (D)–(F) winding height of 6 mm. The columns correspond to coil designs with target depths of (A),(D) ${d_{1/\sqrt 2 }} \ge 0.49$ cm; (B), (E) ${d_{1/2}} \ge 1.31{\mathrm{cm;}}$and (C), (F) ${d_{1/\sqrt 2 }} \ge 0.79$ cm, matching commercial coils MagVenture B35, B65, and B80, respectively, whose performance is denoted with pink dots for comparison. In this comparison, windings of each coil are not connected and are treated as separate closed loops.

We additionally considered designs assuming that $\alpha = \sqrt 2 $. Figure 8 shows energy versus ${S_{1/\sqrt 2 }}$ curves for target depths ${d_{1/\sqrt 2 }} \ge \left\{ {0.49,{\text{ }}0.65,{\text{ }}0.79} \right\}$ cm, corresponding to the B35, B65, and B80 coils respectively. Same as before, spherical support (i.e. more conformal) and hat coils outperformed square flat fdTMS coils. Figures 7(A)–(C) and 8(A)–(C) display results for wires having 6 mm height, and figures 7(D)–(F) and 8(D)–(F)—for filamentary wires. The filamentary wires always outperformed their corresponding non-zero height wire results, indicating that height of the coil should be minimized to ensure best spread and energy performance, to the extent possible within the constraints of conductive power loss and associated coil heating.

### 3.2. Spread as a function of depth

**Figure 9.**
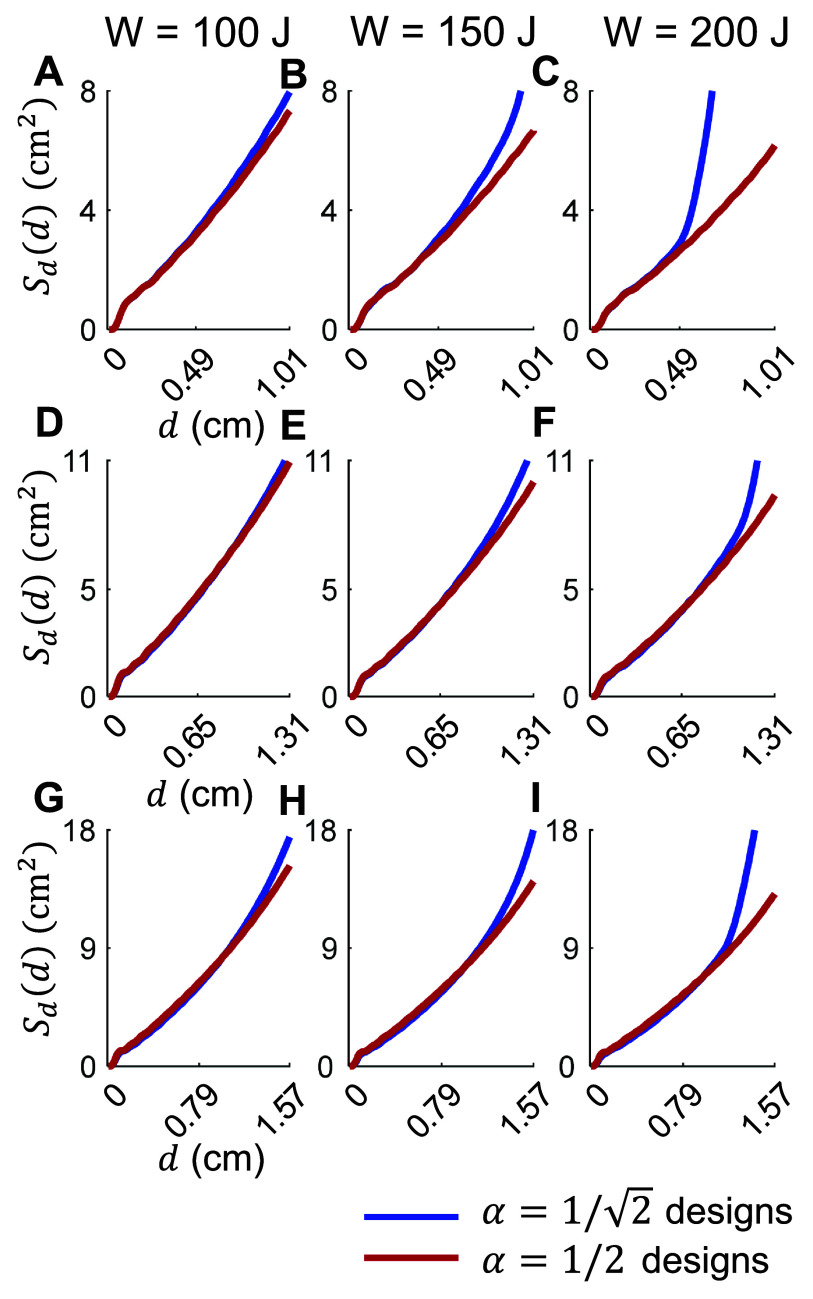
The E-field spread as a function of depth for coils designed with $\alpha = 2$ and $\alpha = \sqrt 2 $. Results are arranged column-wise in increasing energy: (A), (D), and (G) *W* = 100 J; (B), (E), and (H) *W* = 150 J; and (G), (H), and (I) *W* = 200 J. Rows compare fdTMS coils with fixed depth of stimulation (A)–(C) ${d_{1/\sqrt 2 }} \ge 0.49{\text{ cm}}$ and ${d_{1/2}} \ge 1.01{\text{ cm}}$, (D)–(F) ${d_{1/\sqrt 2 }} \ge 0.65{\text{ cm}}$ and ${d_{1/2}} \ge 1.31{\text{ cm}}$, and (G)–(I) ${d_{1/\sqrt 2 }} \ge 0.79{\text{ cm}}$ and ${d_{1/2}} \ge 1.57{\text{ cm}}$.

The amplitude of the coil driving current can be changed to generate a peak cortical E-field that is an arbitrary percentage of the stimulation threshold. For coil design, however, the peak cortical E-field is constrained to $\alpha $ times the E-field strength at the target, ${E_{{\mathrm{TARG}}}}$. Parameter $\alpha $ can be interpreted as the ratio between the maximum induced E-field and the (lower) E-field strength that is still effective for neural stimulation effects. Here we considered the depth–focality trade-off as a function of stimulation depth or, equivalently, as we allow $\alpha $ to vary from $\sqrt 2 $ to 2. Figure 7 shows that designs with $\alpha = \sqrt 2 $ (i.e. peak cortical E-field of $\sqrt 2 {E_{{\mathrm{TARG}}}}$) and $\alpha = 2$ (i.e. peak cortical E-field of $2{E_{{\mathrm{TARG}}}}$) had similar performance for shallow depths. In contrast, the fdTMS coils with $\alpha = 2$ significantly outperformed $\alpha = \sqrt 2 $ designs for deeper targets. Furthermore, as the energy of the coils increased, these performance differences became more significant. This is because the fdTMS coils tend to have large regions of their E-field with strength slightly below threshold. As such, when the fdTMS coils are operated with a peak E-field stronger than $\alpha {E_{{\mathrm{TH}}}}$, the stimulated region will be diffuse, reducing focality. Correspondingly, the choice of $\alpha = 2$ is more robust than $\alpha = \sqrt 2 $ even if the coil will be operated to generate a peak cortical E-field less than twice the field strength at the target. We report both ${S_{1/2}}{\text{ }}$ and ${S_{1/\sqrt 2 }}$ because the operating ratio between peak cortical E-field and stimulation threshold depends on stimulator output; importantly, optimizing ${S_{1/2}}{\text{ }}$ produced designs that are also near-optimal under ${S_{1/\sqrt 2 }}{\text{ }}$ across depths (figure 9).

### 3.3. Choosing number of concentric windings

For practical coil drive currents and efficient energy transfer, TMS coils have inductance $L \ge 8.5{ }\mu {\mathrm{H}}$ [59]. Furthermore, to prevent heating and minimize the resistance of the coil, the wire cross-section is $ \unicode{x2A7E} 8{\text{ m}}{{\mathrm{m}}^2}$. The fdTMS coils typically have winding loops that concentrate in the coil center; as such, multiple layers must be used to achieve a high enough inductance.

**Figure 10.**
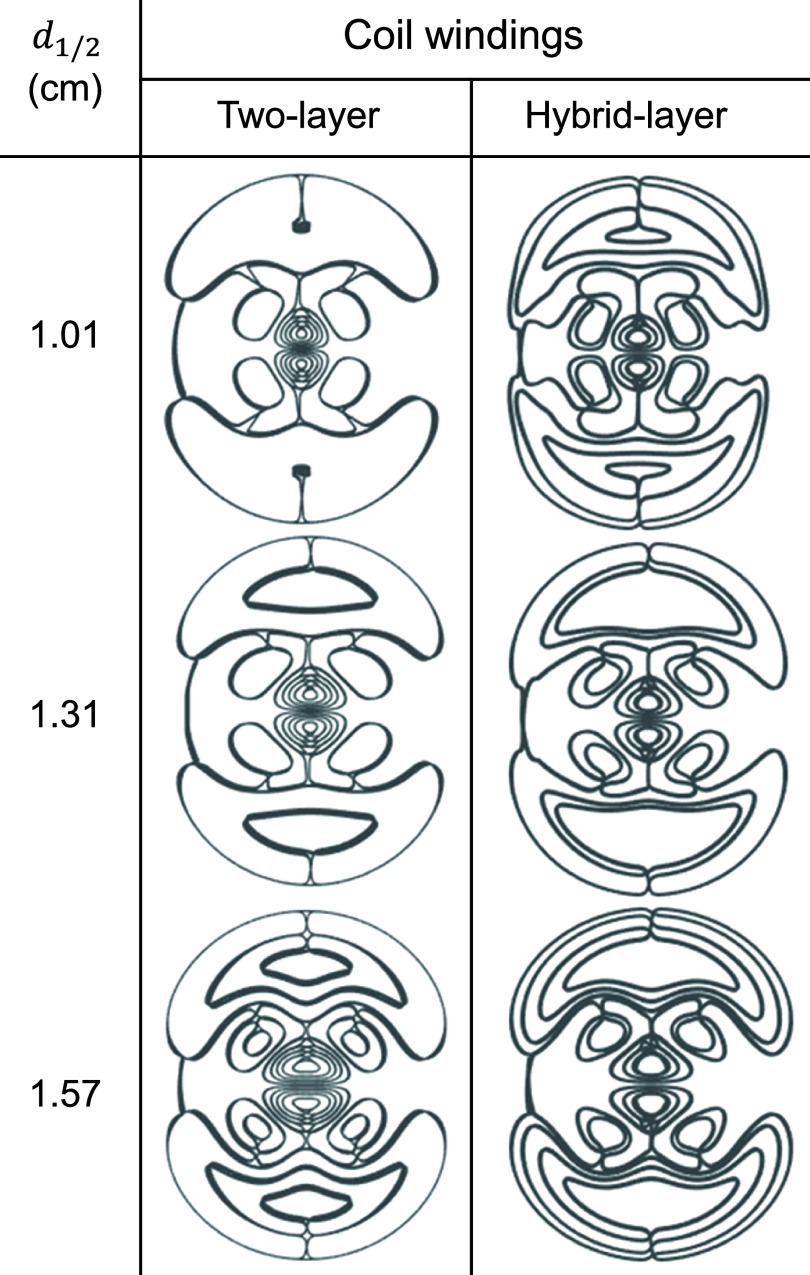
fdTMS coil designs with two-layer and hybrid-layer (three layers in the center, one in the periphery) windings.

We considered two implementations of the fdTMS coils: one using two layers and one using hybrid layers (figure 10). The two-layer implementation had 2.0 mm wide × 4.0 mm tall wire cross-section and 5 concentric loops (i.e. $M = 10$) when discretizing the stream function. This was the maximum number of concentric loops that could accommodate 2 mm wide wire. The hybrid-layer approach used three layers to implement the ‘figure-8’ winding in the coil center and one layer for all other windings, with a 3.0 mm by 3.0 mm wire cross-section. Table 1 summarizes key parameters of the hybrid-layer and two-layer coil designs. The hybrid coils achieved focality that is slightly inferior to the two-layer designs. However, the hybrid-layer designs used less energy than their two-layer counterparts (the energy limit target was 200 J); therefore, we chose to implement the hybrid-layer coils.

**Table 1.** Performance figures of merit for two-layer and hybrid-layer fdTMS coil implementation approaches.

	Two-layer			Hybrid-layer		
${d_{1/2}}$	$ \unicode{x2A7E} 1.01{\text{ cm}}$	$ \unicode{x2A7E} 1.31{\text{ cm}}$	$ \unicode{x2A7E} 1.57{\text{ cm}}$	$ \unicode{x2A7E} 1.01{\text{ cm}}$	$ \unicode{x2A7E} 1.31{\text{ cm}}$	$ \unicode{x2A7E} 1.57{\text{ cm}}$
${S_{1/2}}$	$5.9{\text{ c}}{{\mathrm{m}}^2}$	$9.23{\text{ c}}{{\mathrm{m}}^2}$	$13.2{\text{ c}}{{\mathrm{m}}^2}$	$6.3{\text{ c}}{{\mathrm{m}}^2}$	$9.7{\text{ c}}{{\mathrm{m}}^2}$	$14.4{\text{ c}}{{\mathrm{m}}^2}$
$W$	$215$ J	$197$ J	$197$ J	$168$ J	$163$ J	$169$ J
$L$	$8.1{ }\,\mu {\mathrm{H}}$	$11.9{ }\,\mu {\mathrm{H}}$	$20.5{ }\,\mu {\mathrm{H}}$	$8.8{ }\,\mu {\mathrm{H}}$	$8.8{ }\,\mu {\mathrm{H}}$	$13.5{ }\,\mu {\mathrm{H}}$

### 3.4. E-field characterization

**Figure 11.**
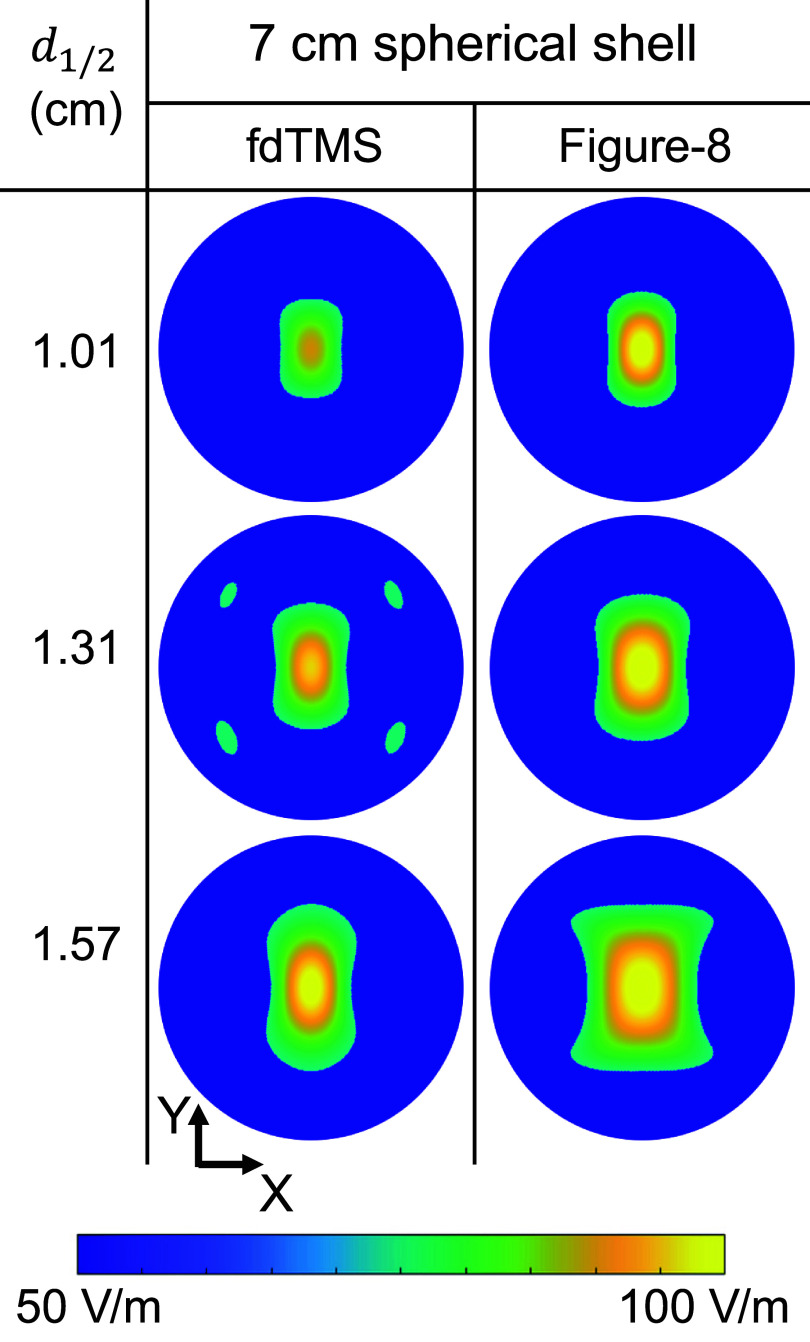
Coil E-field distribution on a 7 cm hemispherical shell for fdTMS and conventional coils.

**Figure 12.**
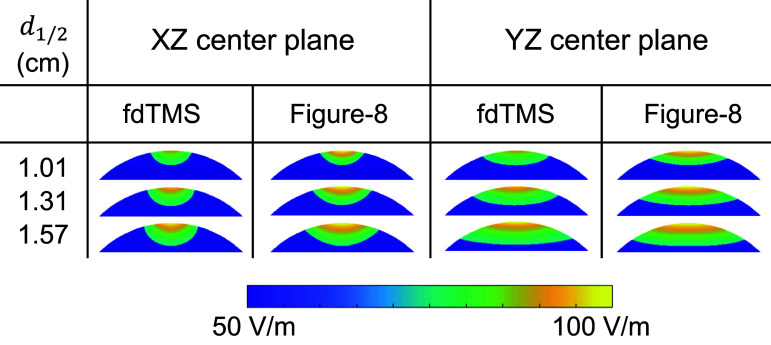
Coil E-field distribution on center cut planes for fdTMS and conventional coils.

Figures 11 and 12 show the E-field distribution for conventional figure-8 coils and fdTMS coils. For the fdTMS coils, the region with E-field exceeding that of the target is more compact than that of the figure-8 coils for matched depth. Additionally, in figure 12 the region above threshold for the fdTMS coil as it penetrates the head appears more compact and cylindrical than that of the figure-8 coils. These results indicate that the lower ${S_{1/2}}$ of the fdTMS coils does result in a more compact E-field distribution.

**Figure 13.**
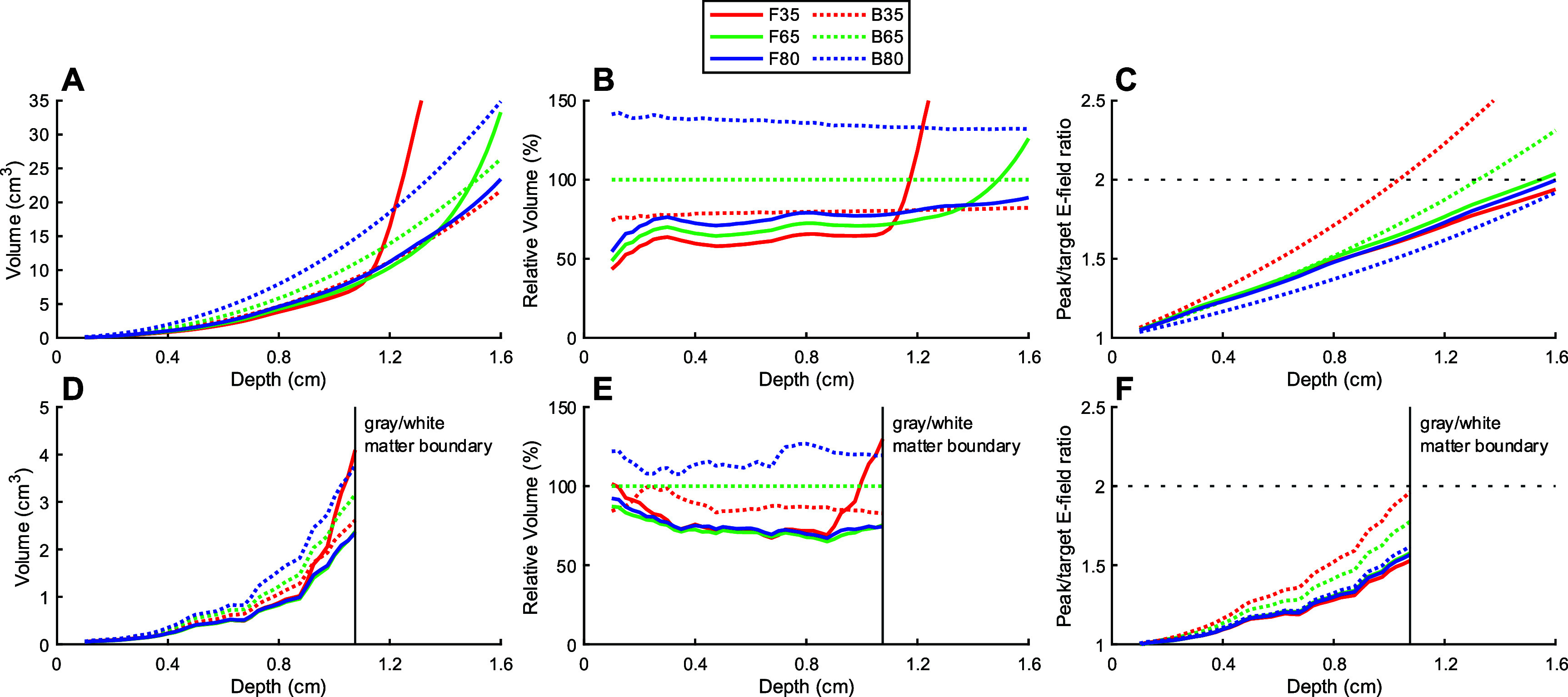
E-field distribution figures of merit versus target depth. (A), (D) Total stimulated volume, (B), (E) stimulated volume relative to the B65 coil, and (C), (F) peak E-field in the brain relative to the E-field at the target at various depths in the brain in the spherical (A)–(C) and realistic (D)–(F) head models. The depth corresponding to the intersection between the horizonal gray line and a curve in (C) and (F) is the value of the ${d_{1/2}}$ figure of merit.

For the sake of simplicity, we shall refer to the hybrid-layer fdTMS ${d_{1/2}} \unicode{x2A7E} 1.01$, ${d_{1/2}} \unicode{x2A7E} 1.31$, ${d_{1/2}} \unicode{x2A7E} 1.57$ coils as F35, F65, and F80, and the corresponding figure-8 coils by their commercial names of B35, B65, and B80 in the following analyses. Figure 13 shows the suprathreshold stimulation ($E &gt; {E_{{\mathrm{TARG}}}}$) volume and peak E-field in the brain versus depth for fdTMS and figure-8 coils in the spherical and realistic head models as computed through finite element analysis. In the spherical model, the fdTMS coils stimulated a smaller volume and are therefore more focal than their conventional counterparts for cortical targets at or closer to the surface than the ${d_{1/2}}$ value, with all fdTMS coils being more focal than their conventional counterparts for target stimulation depths of less than about 1.1 cm in the brain (figures 13(A) and (B)). Furthermore, none of the three fdTMS coils would induce an E-field in the brain that was above twice the target threshold for targets 1.5 cm or closer to the surface (figure 13(C)).

In the realistic head model, the TMS coil center was slightly offset from the hand knob on the vertex of the precentral gyrus to reflect conventional TMS coil placement for motor cortex activation [49]. Consequently, we could simulate the E-field in gray matter up to 1.1 cm deep along the *z*-axis of the coil before reaching the white matter (figure 5). Between depths of 0.2 and 0.9 cm—the approximate depth of stimulation by TMS [49]—the fdTMS coils generally exhibited better focality than the conventional coils (figures 13(D) and (E)) following similar trends as in the spherical head model (figures 13(A) and (B)). The spread of the F35 rapidly increased at greater depths, whereas the other two fdTMS coils (F65 and F80) remained more focal than all three conventional coils. To stimulate a target in the gray matter, none of the coils would induce an E-field in the brain that is above twice that at the target for depths of at least 1.1 cm, approximately at the gray matter/white matter interface (figure 13(f)).

**Figure 14.**
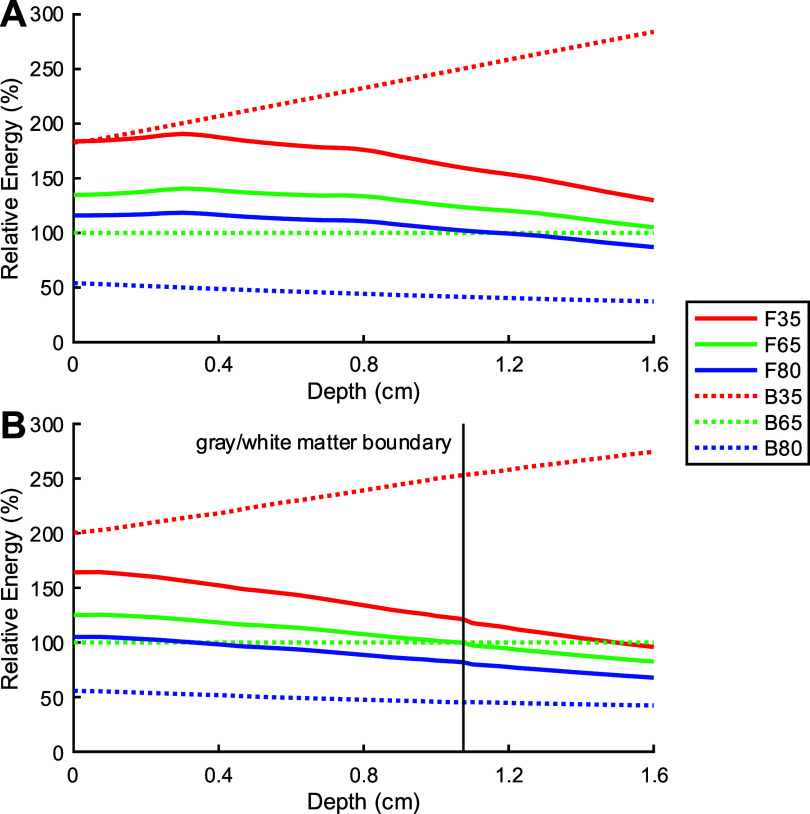
Energy of coils relative to the B65 coil in the (A) spherical and (B) realistic head models.

Figure 14 shows the coil energy relative to the B65 as a function of depth. While the energy of the fdTMS coils generally fell between the B35 and B65 figure-8 coils for superficial targets, the energy relative to the B65 coil decreased as target depth increases. The B35 coil and its fdTMS counterpart required similar energies to reach the brain surface, and the other two fdTMS coils had lower energies, though no lower than that of the B65. Simulations in the realistic head model yielded similar patterns.

**Figure 15.**
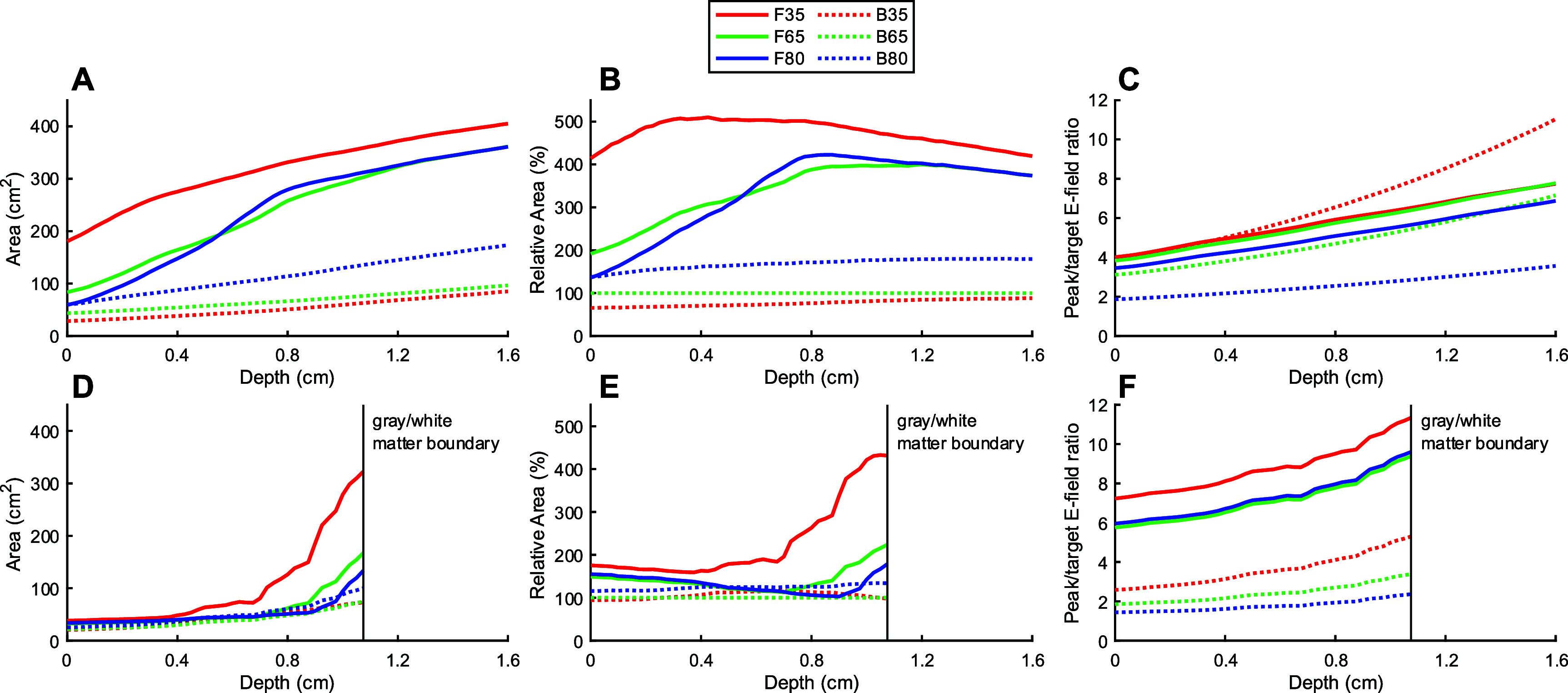
Characterization of scalp stimulation. (A), (D) Total stimulated scalp surface area, (B), (E) stimulated scalp surface area relative to the figure-8 ${d_{1/2}} \ge 1.31$ cm coil, and (C), (F) peak E-field on the scalp relative to the E-field at the target at various depths in the brain in the spherical (A)–(C) and realistic (D)–(F) head models.

TMS coils generate strong E-fields that are known to cause scalp sensations during stimulation. Figure 15 shows the scalp surface area stimulated above the target E-field and peak E-field relative to the target E-field. In the spherical head model, the fdTMS coils stimulated a significantly larger region of the scalp compared to the figure-8 coils (figures 15(A) and (B)) and induced an E-field on the scalp generally between those of the B35 and B65 coils (figure 15(C)).

In the realistic head model, F35 also caused more widespread scalp stimulation, especially for deeper targets in the gray matter. The areas that F65 and F80 stimulated, in contrast to the simulation in the spherical head model, were closer to those for the figure-8 coils (figures 15(D) and (E)). The peak intensity in the scalp for all fdTMS coils was also notably stronger than for any of the figure-8 coils (figure 15(F)). Based on the simulations, this increased peak intensity and extent of scalp stimulation with fdTMS coils could result in more discomfort, which was confirmed in the experimental study with human subjects reported below.

### 3.5. Measurement and validation of implemented coils

**Figure 16.**
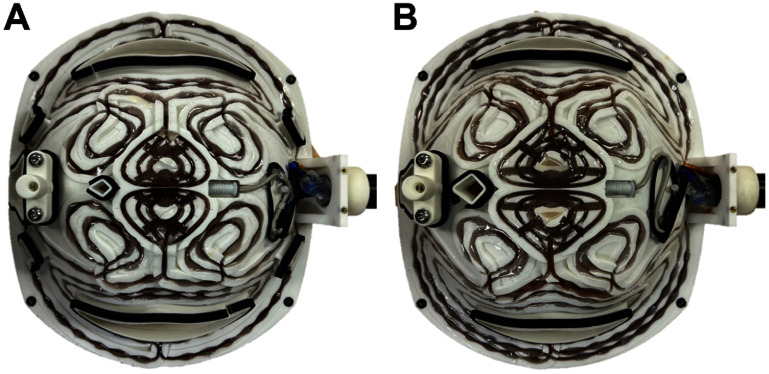
Hardware implementation of the hybrid-layer fdTMS coils with (A) ${d_{1/2}} \ge 1.31$ cm (F65) and (B) ${d_{1/2}} \ge 1.57$ cm (F80) and winding patterns shown in figure 10. In addition to the windings, the coil formers house a neuronavigation tracker pedestal on the left and a temperature sensor (gray) and threaded handle base on the right.

Ultimately, we implemented only the F65 and F80 coils, since our simulation evaluation indicated that the focality benefits of the ${\text{ }}$ F35 design is restricted to superficial layers while requiring more energy and likely causing more scalp discomfort. Figure 16 shows the final fdTMS coil implementations using the respective winding designs from figure 10.

**Figure 17.**
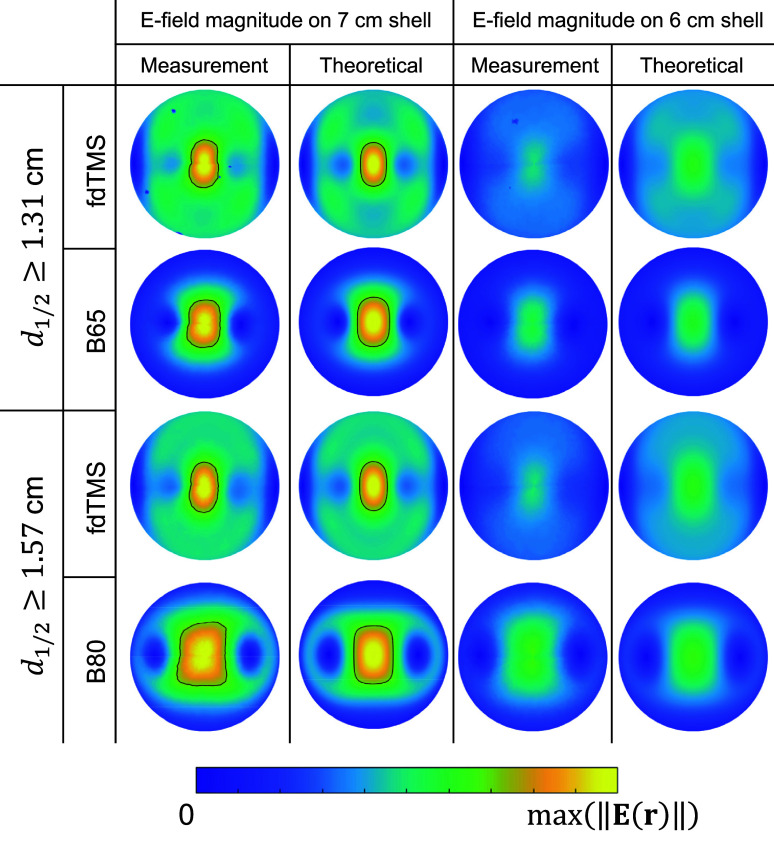
Coil E-field measurements on 7 and 6 cm hemispherical shells compared to theoretical E-field calculations. The black outline encloses the region with E-field strength above $\sqrt 2 $ of the maximum E-field strength.

The E-fields on 7 cm and 6 cm hemispherical shells were measured for the F65 and F80 coils as well as the commercial MagVenture Cool-B65 and B80 coils, which have the same respective depth figures of merit. The E-field measurement results are shown in figure 17, indicating that the simulated and measured E-fields match well on both shells, validating the coil implementation. Furthermore, the contour enclosing the region with an E-field strength above $\sqrt 2 {\text{ }}$ times its maximum was smaller for the fdTMS coils compared to their depth-matched figure-8 counterparts, demonstrating that the fdTMS coils were more focal.

**Figure 18.**
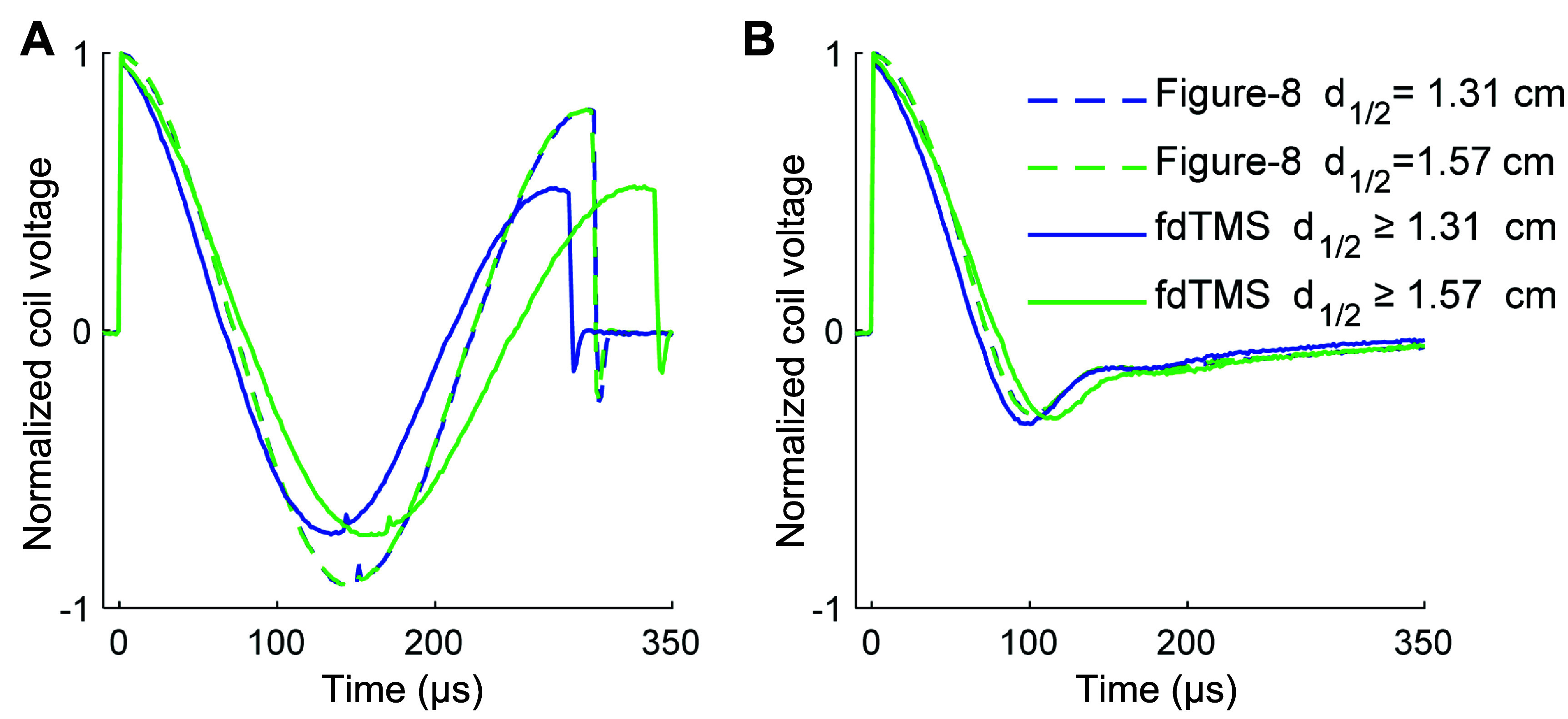
E-field pulse waveforms recorded from the experimental F65 and F80 coils and their figure-8 coil counterparts, MagVenture B65 and B80, respectively. Coils are driven with MagPro X100 with MagOption in standard pulse mode with (A) biphasic and (B) monophasic pulses.

Figure 18 shows the E-field pulse waveforms measured under the coil center for the experimental fdTMS coils and their conventional figure-8 counterparts. The pulse duration of both monophasic and biphasic pulses was comparable across the coils, due to the similar coil inductances. The fdTMS coils had more electrical damping, as evidenced by the lower ending amplitude of the biphasic pulses. Specifically, the energy loss is 77% and 76% for the F65 and F80 coils, compared to 38% and 37% for the MagVenture B65 and B80, respectively. The higher energy loss is a consequence of the larger number of windings in the fdTMS coils, resulting in longer current path through the winding wire, as well as the constraints on the wire diameter to accommodate the dense winding patterns in the center of the coil.

We also measured coil inductance and resistance as summarized in table 2. The fdTMS coil inductances are comparable to the commercial coils. With single pulses at maximum stimulator intensity of the MagPro X100 in power mode, peak pulse current was <10 kA without any observed damage to the coil. Due to their higher resistance than the commercial coils, the fdTMS coils would heat up after long sessions. To mitigate the risk of overheating a coil during an experimental session, we pre-cooled the coils with an ice pack.

**Table 2.** Measured coil inductance and resistance of the commercial (B35, B65, B80) and experimental (F65 and F80) coils.

Coil	B35	B65	B80	F65	F80
Inductance (*μ*H)	12.8	11.8	11.6	10.4	14.9
Resistance (mΩ)	15	11	12	39	48

### 3.6. Evaluation in healthy human subjects

*Participants, feasibility, and safety:* twelve subjects were recruited, of which nine (2 female, 7 male, age: 23.0 ± 2.7, 20–29 years (mean ± standard deviation, range), all right-handed) completed the full study under the final experimental protocol. Of the rest, one withdrew due to difficulty tolerating TMS with the F65 coil, one was excluded due to large hair thickness that made finding RMT impossible with all but the B65 coil, and one was excluded due to challenges fitting the fdTMS coils tangentially to the scalp due to the head shape. No unexpected or serious adverse events occurred during the study. The reported side effects at the end of the TMS session included mild headache (3 subjects), neck pain (1 subject), and jaw pain (1 subject). These side effects are common for TMS procedures [51].

**Figure 19.**
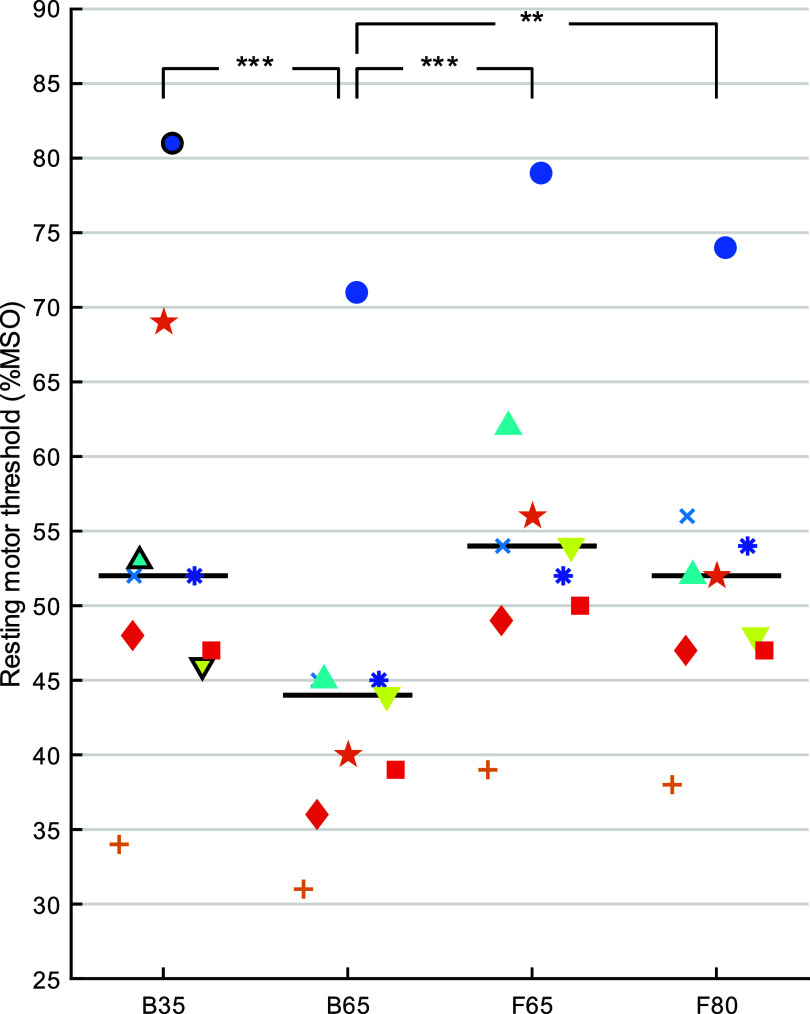
FDI resting motor threshold (RMT) across coils (B35, B65, F65, F80) and subjects. Each symbol corresponds to a subject. All RMTs are determined with MagPro power-mode monophasic pulses, except for the ones in bold which correspond to biphasic pulses. Horizontal lines denote median. ** *p* < 0.01, *** *p* < 0.001.

*Motor threshold:* the RMT varied significantly across coils (*F*_3,24_ = 13.9, *p* < 0.0001), with the RMT of B65 lower than B35, F65, and F80 (*p’s* < 0.002) and the latter three did not differ significantly (figure 19). The significance of the comparisons was not affected by excluding from the analysis the B35 RMTs obtained with a biphasic waveform as opposed to the default power-mode monophasic waveform (*F*_3,21.0_ = 13.9, *p* < 0.0001; post-hoc *p* < 0.002). Since the coil energy is proportional to the RMT squared, B65 required significantly less energy for stimulation than the other coils.

*Extent of muscle activation:* at 100% of FDI RMT, the FDI MEP amplitude did not differ significantly across coils (*F*_3,447_ = 0.818, *p* = 0.484). This supports the effectiveness of the motor thresholding procedure to match the activation produced by the different coils.

**Figure 20.**
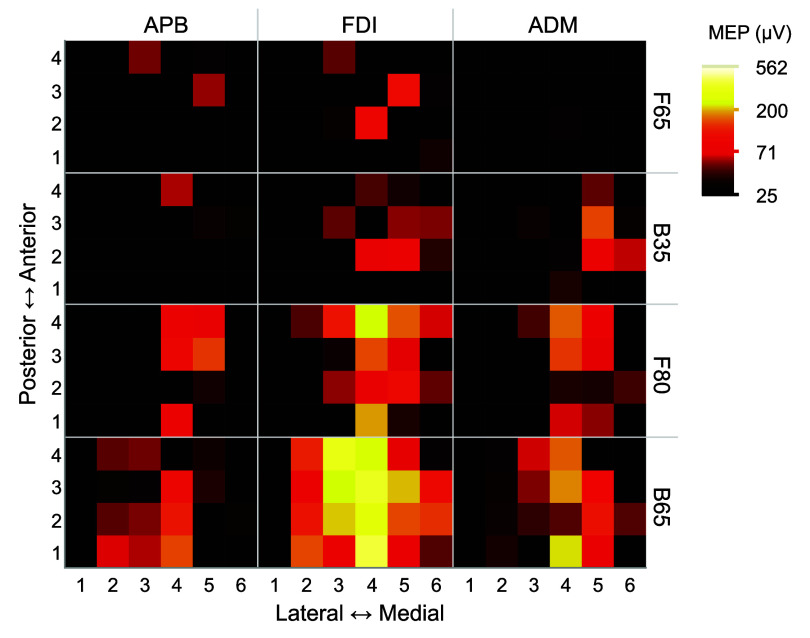
Average MEP peak-to-peak amplitude across mapping grid positions for the different coils (B35, B65, F65, F80) and muscles (APB, FDI, ADM). Median across subjects (*N* = 9) is shown. The axes indicate the lateral-medial and posterior-anterior orientation of the grid over the hand knob of primary motor cortex. The grid step size is 5 mm.

**Figure 21.**
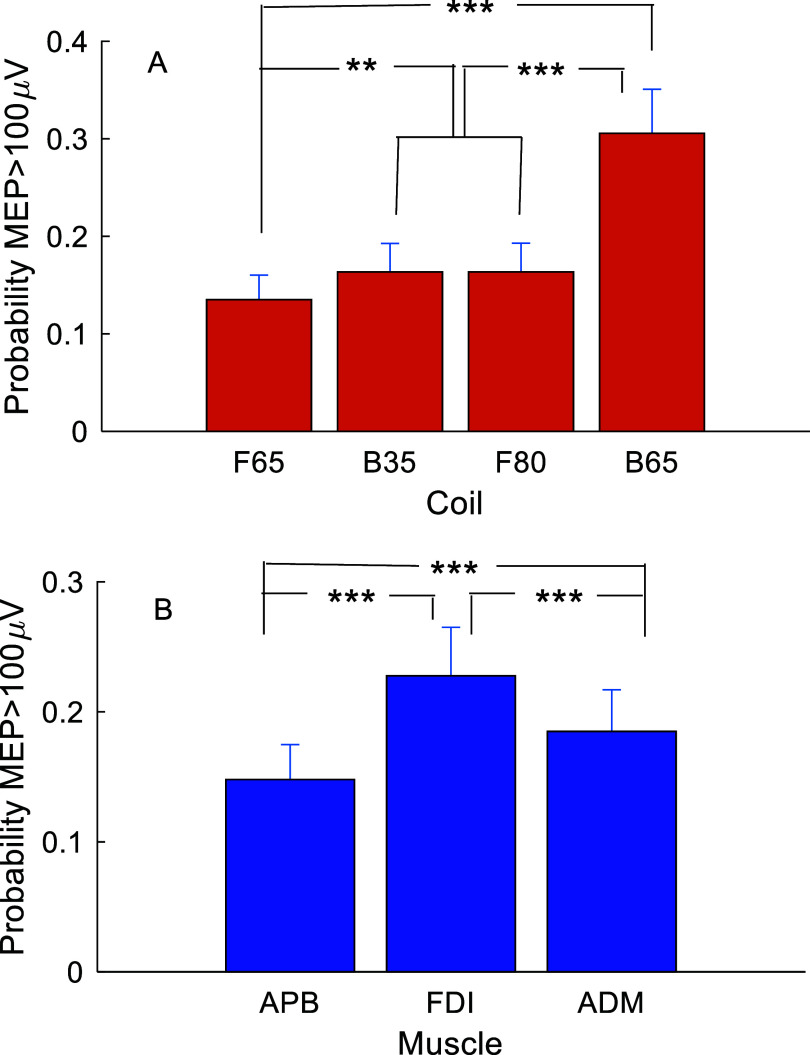
Muscle recruitment across (A) coils and (B) muscles during mapping at 105% FDI RMT. Shown are mean and standard error of probabilities of MEP ⩾ 100 *μ*V estimated by the generalized linear mixed effects model. ** *p* < 0.01, *** *p* < 0.001.

Figure 20 summarizes the average MEP sizes across the mapping grid for the four coils and three muscles at 105% of FDI RMT. The maps suggest that F65 is most focal, followed by B35, F80, and B65. Indeed, the probability of evoking robustly suprathreshold MEPs (peak-to-peak amplitude ⩾ 100 *μ*V) was significantly affected by coil (*F*_3,12 924_ = 114, *p* < 0.0001) and muscle (*F*_2,12 924_ = 43.2, *p* < 0.0001), without a significant interaction between the two (*F*_6,12 924_ = 1.56, *p* = 0.156) (figure 21). The post-hoc tests indicated that F65 had significantly lower suprathreshold MEP probability than the other coils (*t* > 3.22, *p* < 0.0069 for all) and that B35 and F80 had a significantly lower suprathreshold MEP probability than B65 (*t* > 13.1, *p* < 0.0001). FDI had the highest suprathreshold MEP probability, followed by ADM and APB (*t* > 4.5, *p* < 0.0001).

**Figure 22.**
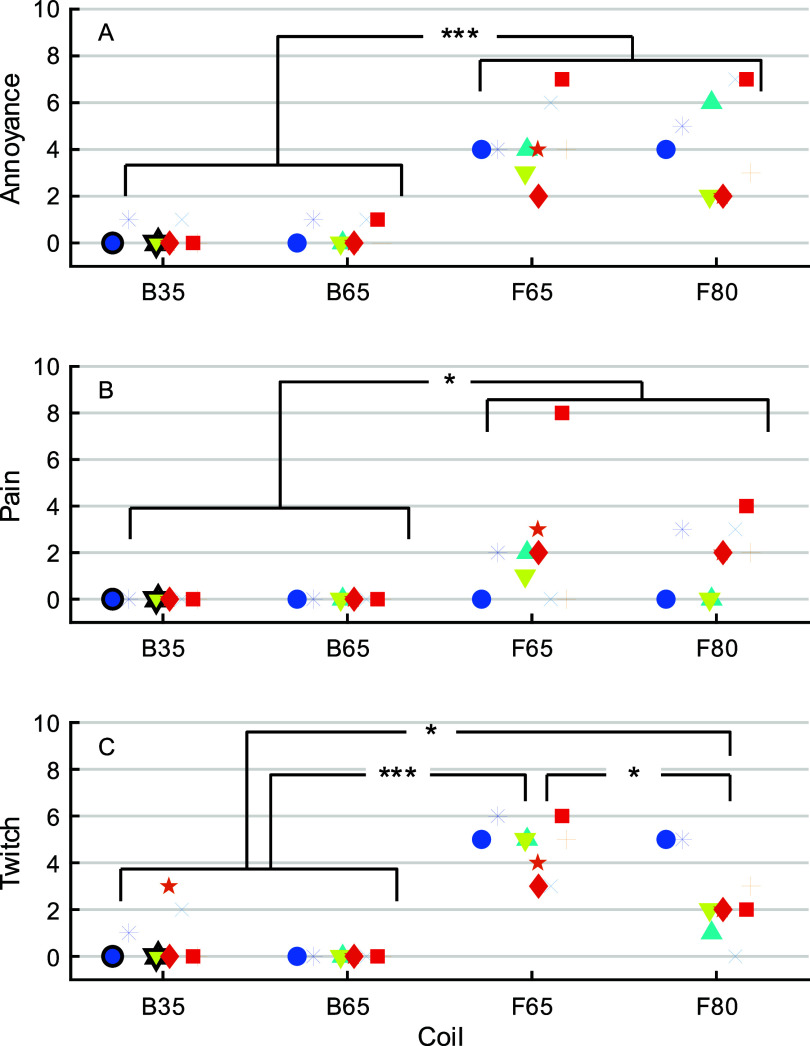
Coil sensation ratings obtained after the mapping at 105% RMT: (A) annoyance (0 = ‘not at all’, 10 = ‘highly annoying’), (B) pain (0 = ‘no pain at all’, 10 = ‘the most pain I could tolerate in this experiment’), and (C) muscle twitching (0 = ‘no twitches’, 10 = ‘a very strong cramp’). Marker convention same as in figure 19. * *p* = 0.05, ** *p* < 0.01, *** *p* < 0.001.

**Figure 23.**
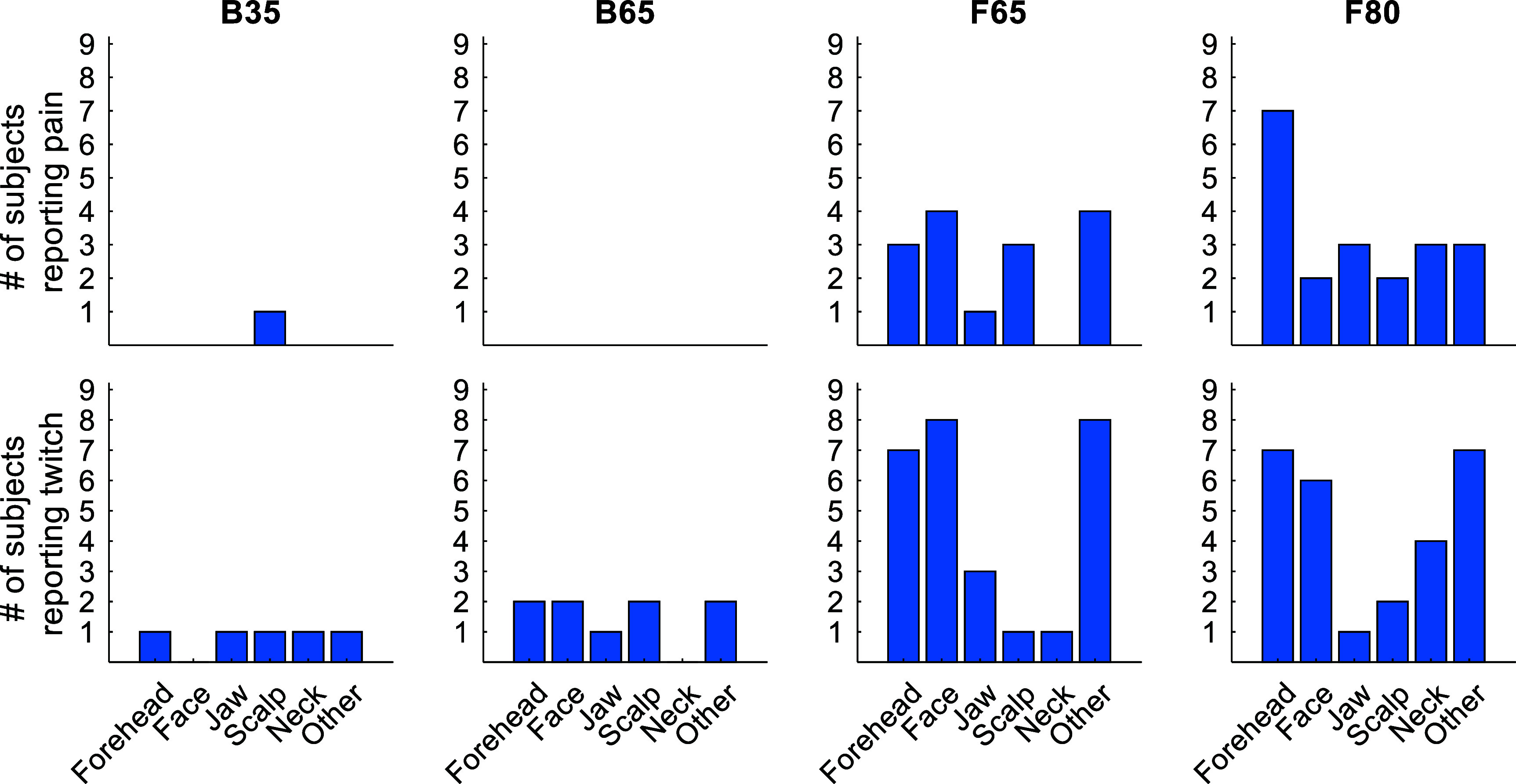
Number of subjects reporting pain (top) or muscle twitches (bottom) for various locations on and around the head. ‘Other’ for pain included eye area, nose, teeth, and gums. ‘Other’ for muscle twitches included eye area, blinking, shoulder, left arm, and cheek.

*Sensation:* Coil type significantly affected the ratings for annoyance (*F*_3,24_ = 47.4, *p* < 0.0001), pain (*F*_3,24_ = 6.06, *p* = 0.0032), and muscle twitching (*F*_3,24_ = 30.0, *p* < 0.0001) (figure 22). The reported locations of the pain and twitching are summarized in figure 23. Across all scales, F65 and F80 received significantly higher ratings than B35 and B65 (*p* < 0.044). F65 and F80 were perceived as equally annoying and painful, but F65 was judged to produce more muscle twitching than F80 (*p* = 0.0020). There were no significant differences between B35 and B65 for any scale.

## 4. Discussion

We introduced a practical method for designing and fabricating fdTMS coils and validating these designs with experimental E-field measurements. The design framework consisted of computational optimization of the winding pattern, an energy-efficient curved ‘hat’ coil surface enabling a wide range of placements, and a hybrid winding approach that improves energy efficiency by using multi-layer windings in some regions of the coil and a single layer in others. The E-field calculations, simulations, and measurements indicated that the fdTMS coils are 19%–33% more focal than their depth-matched figure-8 coil counterparts at their respective target depths. The hybrid layer fdTMS coils achieved increased focality of 24%–27% in a range of depths of 1.31–1.57 cm. For a given coil, as the current increased, the depth of stimulation also increased and focality decreased. The optimized fdTMS designs achieved greater focality than figure-8 coils at all stimulation depths shallower than the target depth. But the improvement in focality led to a wider-range of coil driving current levels. Although focality evaluations in the realistic head model are limited by the lack of variation, concordance with the experimental results, which captured individual variation not only of head anatomy but also neurophysiology, suggest that the modeling results are representative of motor activation. The experimental motor mapping results as well as the significant difference in suprathreshold MEP probability at 105% RMT indicated differences in the extent of neural activation, and hence focality, across coils. Among the coils used in the study, F65 was most focal, followed by B35, F80, and B65.

The design of focal TMS coils involved trade-offs between focality, depth, and energy. This is illustrated by the physics of TMS induced E-fields in a homogenous spherical head model [11]. Equation (13) in Koponen *et al* [23] indicates that as the order of spherical harmonics on a spherical shell is increased, the decay will increase geometrically. In other words, sharp variations in the E-field on a spherical shell will result in rapidly decaying E-fields into the head. Furthermore, since higher order harmonics decay rapidly into the head, all of their associated energy is localized at shallow depths. And because the induced E-field decays rapidly with distance (high-order falloff), producing stronger, more spatially confined E-fields at greater depth demands substantially more energy. These energy requirements, together with concerns about scalp tolerability, made it impractical to pursue F35—the fdTMS analog of the highly focal B35 coil. Nevertheless, the less-focal fdTMS design, F65, was still more focal than B35 in both E-field simulations and human motor mapping.

The fdTMS coils produced a broader E-field distribution on the scalp than standard coils, and stimulated a larger region of scalp tissue. One reason for this spread is that the fdTMS coils have large ‘biasing’ loops, which increase the E-field of more brain regions to near threshold. The E-fields associated with these ‘biasing’ loops were stronger on the surface of the scalp so that broad regions of scalp were exposed to stronger E-fields. As anticipated by the models of the E-field in the scalp, the tolerability of the fdTMS coils (F65 and F80) was markedly worse than that of the conventional coils (B35 and B65) with respect to reported annoyance, pain, and muscle twitching. While tolerability of the developed focal coils is an issue for stronger suprathreshold stimulation or various more painful targets compared to conventional coils, they can be used, for example, with subthreshold and low-strength, near-threshold magnetic stimulation paradigms which do not generate a scalp sensation [60].

The fdTMS coils exhibit higher resistive losses than conventional designs due to the presence of additional loops and tight winding packing near the coil center, which increases the total wire length and limits the wire diameter to 2.05 mm. Optimization strategies that explicitly minimize resistive loss or power dissipation typically favor larger, more spatially distributed windings, which are incompatible with achieving high focality at depth. Accordingly, in this work dissipation-related factors—such as wire cross-section, resistance, and operational limits—are treated as feasibility constraints, while the optimization itself targets the depth–focality–energy trade-off. Nonetheless, TMS pulse durations are well below any conventional ampacity formulas [23, 61]. These levels of power loss are acceptable for single-pulse applications such as mapping, where monophasic pulses are commonly used. For rapid-rate repetitive TMS applications, where biphasic pulses are typically used and pulse energy recovery is important, the fdTMS coils could be redesigned with fewer higher-order modes or quantized with larger cross section and/or parallel branches to reduce the energy loss and heating, which may result in reduction in their focality advantages. Alternatively, air- or liquid-cooling in combination with a more powerful TMS device can enable the necessary power. Notably, even with additional energy requirements and reduced wire-cross section, experimental evidence suggests that for the most common clinical paradigm with 20 Hz rTMS, the power requirements of fdTMS coils could be achieved by the TMS device used in this study [23, 61, 62].

We also saw coil fitting issues during the experimental portion of the study. Future designs should consider an inner coil surface that would account for a wider population of head shapes and sizes, which will result in reduced curvature of the coil and better fitting. Furthermore, the coil periphery and handle could be designed to minimize collisions with the subject’s shoulders. In comparison, the conventional B80 coil, whose loops are bent at 120°, proved less practical for hand motor–area mapping than the hat-shaped fdTMS coils.

## 5. Conclusions

The fdTMS coil designs developed in this paper optimized focality within the constraints of brain stimulation by magnetic induction including limitations on the E-field depth, pulse energy, coil size, and winding density. These coils are more focal than their depth-matched figure-8 coil counterparts, and we believe these improvements represent the practical limits of E-field focality of TMS. The main limitations of the fdTMS coils are more discomfort associated with stronger and broader scalp stimulation as well as increased energy loss and associated heating. Overall, the presented design framework pushed the practical limits of TMS coil focality and identified potential advantages as well as challenges for future fdTMS applications.

## Data Availability

The data that support the findings of this study are openly available at the following URL/DOI: https://github.com/luisgo/fdTMS [63].
